# The primate gut microbiota contributes to interspecific differences in host metabolism

**DOI:** 10.1099/mgen.0.001322

**Published:** 2024-12-02

**Authors:** Elizabeth K. Mallott, Sahana Kuthyar, Won Lee, Derek Reiman, Hongmei Jiang, Sriram Chitta, E. Alexandria Waters, Brian T. Layden, Ronen Sumagin, Laura D. Manzanares, Guan-Yu Yang, Maria Luisa Savo Sardaro, Stanton Gray, Lawrence E. Williams, Yang Dai, James P. Curley, Chad R. Haney, Emma R. Liechty, Christopher W. Kuzawa, Katherine R. Amato

**Affiliations:** 1 Department of Anthropology, Northwestern University, Evanston, IL, USA; 2 Department of Biology, Washington University, St. Louis, MO, USA; 3 Department of Biological Sciences, University of California, San Diego, CA, USA; 4 Department of Psychology, University of Texas, Austin, TX, USA; 5 Department of In Vivo Pharmacology Services, The Jackson Laboratory, Sacramento, CA, USA; 6 Department of Bioengineering, University of Illinois, Chicago, IL, USA; 7 Toyota Technological Institute, Chicago, IL 60637, USA; 8 Department of Statistics and Data Science, Northwestern University, Evanston, IL, USA; 9 Department of Comparative Medicine, The University of Texas MD Anderson Cancer Center, Bastrop, TX, USA; 10 Center for Advanced Molecular Imaging, Northwestern University, Evanston, IL, USA; 11 Department of Endocrinology, Diabetes, and Metabolism, College of Medicine, University of Illinois, Chicago, IL, USA; 12 Department of Pathology, Feinberg School of Medicine, Northwestern University, Chicago, IL, USA; 13 Center for Comparative Medicine, Northwestern University, Chicago, IL, USA

**Keywords:** encephalization, gluconeogenesis, life history, physiology, short-chain fatty acids (SCFAs)

## Abstract

Because large brains are energetically expensive, they are associated with metabolic traits that facilitate energy availability across vertebrates. However, the biological underpinnings driving these traits are not known. Given its role in regulating host metabolism in disease studies, we hypothesized that the gut microbiome contributes to variation in normal cross-vertebrate species differences in metabolism, including those associated with the brain’s energetic requirements. By inoculating germ-free mice with the gut microbiota (GM) of three primate species – two with relatively larger brains and one with a smaller brain – we demonstrated that the GM of larger-brained primates shifts host metabolism towards energy use and production, while that of smaller-brained primates stimulates energy storage in adipose tissues. Our findings establish a causal role of the GM in normal cross-host species differences in metabolism associated with relative brain size and suggest that the GM may have been an important facilitator of metabolic changes during human evolution that supported encephalization.

**Impact Statement**

While there are many studies exploring the role of the gut microbiota (GM) in shaping metabolism within a given host species, studies examining gut microbial influences on normal differences in metabolism across host species are limited. We used a germ-free mouse model to show that the GM of different primate species drives differences in host metabolism. Furthermore, the GM of relatively large-brained primate species shifts host metabolism towards energy production and utilization, while the GM of smaller-brained primate species shifts host metabolism towards energy storage. Metabolic shifts towards energy production and utilization are believed to underlie the evolution of large brains in primates, such as humans. As a result, these data provide preliminary evidence for the role of the GM in human evolution.

## Data Summary

Raw 16S rRNA gene sequences from donor stocks and mouse faecal samples, raw shotgun sequences from mouse faecal samples and mouse liver RNA sequences are available in the Sequence Read Archive (PRJNA1003977). All code used for analysis can be found at https://github.com/Kramato-lab/GF_mouse_primate_23 and https://github.com/Mallott-Lab/PrimateMicrobiomeandBrainGrowth.

## Introduction

The brain is a metabolically expensive tissue to grow and maintain [1,3]. It has a higher rate of glucose metabolism than nearly all other organs in the body to support functions such as neuronal signalling, synapse formation and information processing [4]. The challenges of maintaining the high costs of cerebral tissue are particularly marked in primates, which tend to have large brains relative to their body sizes [5]. Accordingly, primates with higher encephalization quotients (EQs) (i.e. brain size relative to body size) generally have higher fasting blood glucose [6]. Humans, which have the largest relative brain size of all primates, exhibit higher daily total energy expenditure than expected based on body size [7]. Knowledge of the mechanisms that shape these differences in primate metabolism and the extent to which they are linked to differences in encephalization are critical for understanding primate brain development and life history, as well as the evolution of large brains. Nevertheless, systematic data comparing metabolic physiology across primate species are rare, and data describing the mechanisms through which metabolism is shaped are even rarer. Some comparative studies have detected genetic and epigenetic differences among primates with larger and smaller relative brain sizes, but the identified pathways do not clearly link to differences in systemic metabolism [8].

Variation in the gut microbiota (GM) represents an unexplored mechanism through which primate metabolism could facilitate different brain energetic requirements. Studies implicating the GM in metabolic disease have demonstrated strong links between specific microbial functions and host metabolic traits [9,10]. In particular, the short-chain fatty acids (SCFAs), acetate, butyrate and propionate, produced by the GM from the fermentation of fibre and amino acids, serve as energy sources for hosts and also influence host metabolic processes both directly and through SCFA receptor-mediated signalling [11]. These processes include appetite and satiety, lipogenesis, cholesterol and triglyceride synthesis, and glucose–insulin metabolism [9,12, 13]. Although most studied for their role in diseases such as diabetes and obesity, the interactions between SCFAs and these host metabolic processes could also shift systemic metabolism towards increased glucose production to support the high energetic demands of large brains and/or towards increased somatic functions such as growth, maintenance and fat deposition. SCFAs, like other microbial metabolites, may also affect brain physiology by facilitating communication between the gut and brain [14], making them an important target for understanding brain evolution and associated life-history trade-offs more broadly. Comparative SCFA data collected systematically across primate species are limited [15], but GM composition and function have been shown to differ markedly between primate species [16,17]. The extent to which these differences contribute to interspecific variation in primate physiology, including metabolism and life history, is currently unknown. We hypothesize that they play an important role in mediating the metabolic strategies necessary for growing and maintaining a large brain while maintaining other body systems.

Here, we use a mouse model to provide the first evidence that differences in GM function across three primate species are linked to differences in host metabolic phenotypes in ways that are consistent with species differences in brain energetic requirements. We inoculated germ-free mice with the GM of three primate species: macaques (*Macaca mulatta*), squirrel monkeys (*Saimiri boliviensis*) and humans (*Homo sapiens*). Although phylogenetically distantly related (one is a platyrrhine monkey from the Americas and the other an African ape), both humans and squirrel monkeys can be considered ‘brain-prioritizing’ species as they exhibit rapid postnatal brain growth and a relatively encephalized adult state (i.e. are large-brained for their body size), thus requiring more energy to support the brain across the lifecycle [18,19]. In contrast, macaques (catarrhine monkeys from Africa) exhibit slower postnatal brain growth rates and also have a less-encephalized adult state [18,19], thus requiring less energy to support the brain. Therefore, we tested the hypothesis that GMs of brain-prioritizing primates facilitate differences in host metabolic phenotypes that favour increased energy available to the brain.

Our results provide support for our overall hypothesis. Mice with the GMs from the two distantly related primate species with relatively high-EQ had a metabolic phenotype consistent with higher host energy use and production via increased food consumption and gluconeogenesis in the liver. In contrast, mice with the GM from a less-encephalized (low-EQ) primate species exhibited faster weight gain and increased deposition of adipose tissue, providing support for a potential trade-off between glucose use and deposition of adipose tissue [20]. These differences were linked to differences in microbial metabolite concentrations, particularly SCFAs.

## The primate GM causally affects normal host metabolic phenotypes in a pattern consistent with relative encephalization

We inoculated weanling C57BL/6 germ-free mice with the GM of adult macaques (*M. mulatta*; low-EQ), squirrel monkeys (*S. boliviensis*; high-EQ) and humans (*H. sapiens*; high-EQ) for a total of three treatments. Donor faecal sample collection methods varied slightly between humans and non-human primates, as described in the Methods. Mice were maintained with *ad libitum* feeding on a standard chow diet for 60 days, and data describing mouse physiology and GM function were collected at multiple time points (Fig. S1, available in the online Supplementary Material of this article). All primate GMs were successfully transferred to the mice. The GM composition of mouse faecal samples after 3 and 60 days was similar to that of the donor stocks, and there were clear differences in GM composition between treatment groups at both time points (Figs S2, S3). Donor stocks and mouse faecal samples at the first time point shared 18.4, 5.9 and 6.2% of amplicon-sequence variants (ASVs) for humans, macaques and squirrel monkeys, respectively. Sixty-three per cent of the microbial genera present in the donor stocks from humans were also present in mouse faecal samples at the first time point (Table S1). Thirty-seven per cent of macaque genera (Table S2) and 30% of squirrel monkey genera (Table S3) were present in mouse faecal samples at the first time point.

**Fig. 1.**
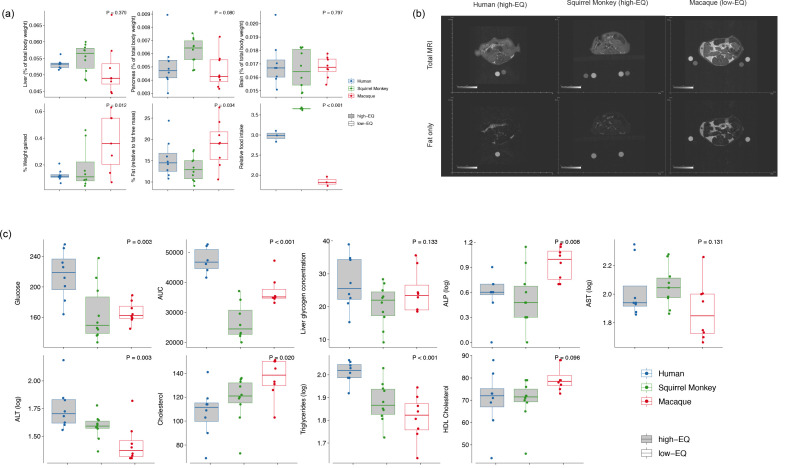
Physiological variables distinguishing mice inoculated with the microbiome of high-EQ and low-EQ primates. Weight gain, per cent fat and average food consumption (measured by cage) differ between high-EQ and low-EQ primate-inoculated mice (**a**). Adiposity, as measured by MRI, differs between high-EQ and low-EQ primate-inoculated mice (**b**). Blood chemistry differs between high-EQ and low-EQ primate-inoculated mice (**c**).

At the 60 day assessment, we detected differences in a range of metabolic traits between mice inoculated with high-EQ primate GMs and mice inoculated with low-EQ primate GMs (Fig. 1). There was no difference in host brain size across treatments (*F*_2,23_ = 0.2, *P* = 0.8; Fig. 1a), which is expected given that brain size is highly genetically canalized across animal species [21]. In other organs, where more plasticity is expected, we observed non-significant trends towards increased liver size (*F*_2,23_ = 1.0, *P* = 0.4) and pancreas size (*F*_2,23_ = 2.8, *P* = 0.08) in high-EQ primate-inoculated mice (Fig. 1a). In contrast, high-EQ primate-inoculated mice exhibited significantly lower per cent body fat (compared to fat-free mass; *F*_2.23_ = 3.9, *P* = 0.03; Fig. 1a, b) and, as a result, also gained less weight (*F*_2,23_ = 5.4, *P* = 0.01; Fig. 1a), despite consuming more food on average (*F*_2,8_ = 370.6, *P* < 0.001; Fig. 1a). Pathological examination of caecal tissue samples from a subset of mice in each treatment group revealed no abnormalities (Fig. S4).

High-EQ primate-inoculated mice also appeared to have heightened energy production. They had increased fasting blood glucose (*F*_2,23_ = 7.5, *P* = 0.003; Fig. 1c), a pattern that was particularly marked in mice that were inoculated with the human GM. There were no differences in liver glycogen content across treatments (*F*_2,23_ = 2.2, *P* > 0.1; Fig. 1b). However, compared to low-EQ primate-inoculated mice, high-EQ primate-inoculated mice exhibited increased blood concentrations of ALP (*F*_2,23_ = 6.1, *P* = 0.008; Fig. 1c) and ALT (*F*_2,23_ = 7.8, *P* = 0.003; Fig. 1c), the latter of which is a key enzyme involved in gluconeogenesis – the production of glucose from other carbohydrates, lipids and proteins. High-EQ primate-inoculated mice also had increased triglycerides (*F*_2,23_ = 12.4, *P* = 0.0002; Fig. 1c) and decreased cholesterol (*F*_2,23_ = 4.6, *P* = 0.02; Fig. 1c). Together, these differences suggest that high-EQ primate-inoculated mice had metabolisms programmed to produce, but not store, higher amounts of glucose, while low-EQ primate-inoculated mice had metabolisms programmed not only to store glucose as fat but also to produce less glucose overall.

## GM composition and functional potential differ between High- and Low-EQ primate-inoculated mice

**Fig. 2.**
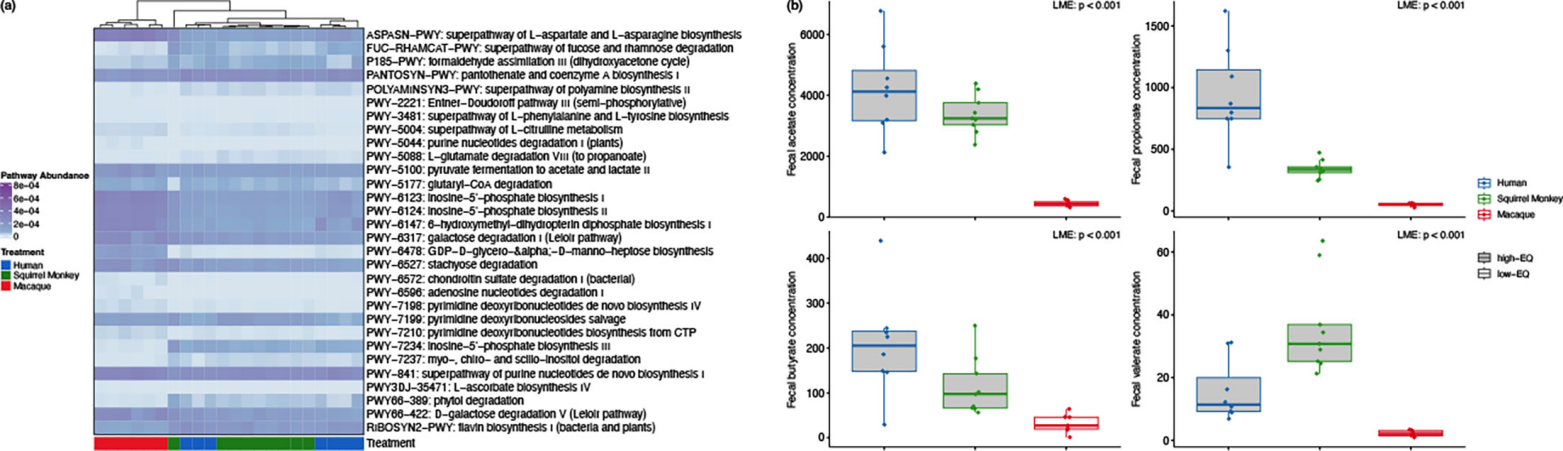
Functional differences between the GM of mice inoculated with high-EQ and low-EQ primate gut microbiomes. The relative abundance of multiple microbial pathways (**a**) differed significantly both between mice inoculated with human and macaque gut microbiomes and between mice inoculated with squirrel monkey and macaque gut microbiomes, but not between mice inoculated with human and squirrel monkey gut microbiomes. Faecal metabolites of SCFAs (**b**) had a higher concentration in samples from mice inoculated with human and squirrel monkey gut microbiomes compared with concentrations in mice inoculated with macaque gut microbiomes.

Overall GM composition and functional potential differed across treatment groups (Figs. S5 and S6). However, only a handful of GM taxa varied across treatments (Figs. S7–9), while GM pathways related to fucose and rhamnose degradation, biosynthesis of amino acids (citrulline, inosine and threonine), pyruvate biosynthesis, the fermentation of pyruvate to acetate and lactate, glycogen degradation, galactose degradation and starch degradation were more abundant in high-EQ primate-inoculated mice than in low-EQ primate-inoculated mice (Fig. 2a). In contrast, pathways related to the fermentation of pyruvate to butanoate, the fermentation of glutamate to propanoate and glycogen synthesis were more abundant in low-EQ primate-inoculated mice (Fig. 2a). Additionally, high-EQ primate-inoculated mice had increased faecal acetate (*F*_2,21_ = 36.9, *P* < 0.001), propionate (*F*_2,21_ = 30.4, *P* < 0.001), butyrate (*F*_2,21_ = 12.0, *P* = 0.0003) and valerate (*F*_1,21_ = 20.4, *P* < 0.001) concentrations (Fig. 2b).

Faecal SCFA concentrations quantify SCFAs that are not absorbed by the host. However, since there are no known consistent differences in absorption rates of different SCFAs by the host, faecal SCFA concentrations are generally believed to be proportional to SCFA production and absorption in the gut [22]. Differences in microbial acetate and propionate production are likely to have important consequences for host metabolism. Both are absorbed in the gut and often reach organs, including the liver, in high concentrations [23,25]. Based primarily on studies in mice, it is known that acetate is a substrate for cholesterol and triglyceride synthesis in the liver, and increased acetate concentrations incite lipogenesis in white adipose tissue [26]. However, increased propionate concentrations block this effect of acetate, and high concentrations of both acetate and propionate trigger gluconeogenesis in the liver as well as in other tissues [27,28]. These effects can be mediated via influences of SCFAs on norepinephrine, glucagon, insulin, ghrelin, neurotransmitters and fatty-acid-binding proteins [29,30]. Therefore, the increased microbial production of acetate and propionate by brain-prioritizing primate GMs could directly result in reduced lipogenesis and increased gluconeogenesis. The glucose produced via this pathway could be a valuable energy source for the brain, particularly during postnatal growth and development when substrate requirements peak [1]. Additionally, butyrate is preferentially used as an energy source by colonocytes [31]. Given that the gut is the most energetically expensive tissue after the brain [3,32], the use of butyrate by the gut could liberate other sources of host energy for the brain. Finally, SCFAs can cross the blood–brain barrier [33] and could be used directly as an energy source or as a metabolic signalling molecule there.

## Liver gene expression differs between High- and Low-EQ primate-inoculated mice

Given patterns in both mouse physiology and GM function that indicated an important role of the liver in mediating the relationship between GM and host metabolism, we used TagSeq to assess differential gene expression in mouse livers at the end of the 60-day experimental period. In addition to donor-species-specific differences in liver gene expression (Fig. S10), we found that high- and low-EQ primate-inoculated mice exhibited distinct liver gene expression patterns (Fig. S11). Some of these genes have been associated with gluconeogenesis, lipogenesis, cholesterol synthesis and various aspects of fatty acid metabolism and adipocyte development (e.g. *Fabp5*, *Fasn*, *Elovl6* and *Mup12*) [34,37]. However, control of liver metabolic processes is not solely driven by variation in gene expression and is also closely linked to patterns of energy and metabolite flux [38,39]. Other key regulatory mechanisms are also likely at play, such as the availability of lactate and alanine as substrates for gluconeogenesis. Both of these substrates can be produced from pyruvate–lactate via pyruvate degradation and alanine via interactions between pyruvate and branched chain fatty acids like valerate. Our GM data provide evidence of increased pyruvate degradation, increased pyruvate biosynthesis and increased valerate concentrations in high-EQ primate-inoculated mice.

**Fig. 3.**
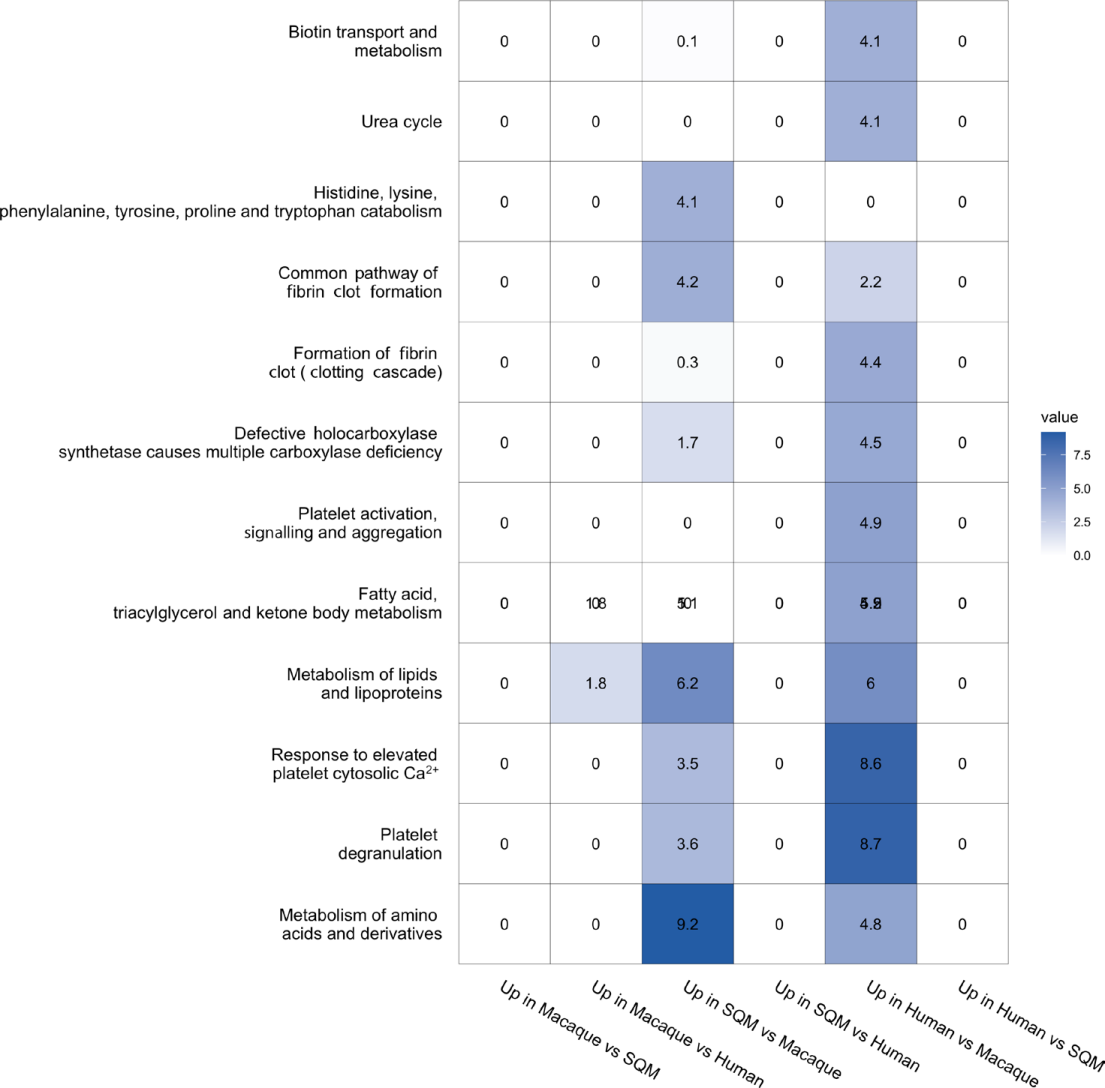
Functional pathways that are differentially expressed in the mouse liver across treatments. Upregulated genes in each pairwise species comparison were identified. Pathway enrichment was performed on each set of differentially expressed genes using the Reactome pathway database. Values and shading represent the negative log-adjusted *P*-value such that more significant enrichments are shaded in darker blue. SQM = squirrel monkey.

Furthermore, MiMeNet analysis showed that 87 microbial ASVs could be used to predict 191 host genes that were differentially expressed in the livers of high-EQ primate-inoculated mice (Fig. S12). Genes predicted by these ASVs were clustered into six different modules, and the two largest modules contained genes associated with fatty acid metabolism and amino acid metabolism, respectively (Fig. S13). Functional analysis of TagSeq data also indicated that high-EQ primate-inoculated mice exhibited enrichment for amino acid, lipid and fatty acid metabolism in the liver as well as altered metabolism and transport of biotin, a key co-factor in many of these pathways (Fig. 3). This pattern provides further evidence suggesting that high-EQ primate GMs programme host metabolism for energy production via gluconeogenesis and other pathways closely tied to lipid signalling. High-EQ primate-inoculated mice were also enriched for platelet signalling and, to some extent, fibrin clot formation despite showing no signs of organ damage (Fig. 3). In addition to their immune roles such as wound healing, platelets are recognized as key calcium-dependent signalling molecules that influence homeostasis and plasticity in the central nervous system, including the brain, by modulating processes including the formation of new neurons [40,41]. Therefore, this suite of metabolic processes may play an important role outside the context of metabolic disease, shifting host metabolism towards energy production to support the brain.

## Humans are distinct from High-EQ squirrel monkeys

In addition to overall differences between high- and low-EQ primate-inoculated mice, mice inoculated with human GMs were distinct in their metabolic physiology and liver gene expression compared to all other mice, consistent with the fact that humans exhibit the highest EQ of all primates. For example, human-inoculated mice exhibited higher fasting glucose (*F*_2,23_ = 7.5, *P* = 0.003; Fig. 1c) and a slower return to baseline fasting glucose levels in response to a 120 min glucose tolerance test compared to all other mice (*F*_2,23_ = 7.53, *P* = 0.003, Fig. 1). Similarly, human-inoculated mice had the highest triglycerides (*F*_2,23_ = 12.4, *P* = 0.0002; Fig. 1c) and lowest cholesterol (*F*_2,23_ = 4.6, *P* = 0.02; Fig. 1c). They also demonstrated the least weight gain during the study (*F*_2,23_ = 5.4, *P* = 0.01). In terms of microbial function, human-inoculated mice exhibited the highest proportions of propionate (*F*_2,21_ = 30.4, *P* < 0.001) and butyrate (*F*_2,21_ = 12.0, *P* = 0.0003). Together, these patterns suggest that the human GM may bias host metabolism away from lipogenesis and towards gluconeogenesis even more than those observed in other primates. Thus, our data indicate a potential role of GM in shaping human metabolic adaptations that facilitate the development and maintenance of large brains.

Overall, our results are consistent with the role of the GM in mediating metabolic phenotypes for energy use and production versus storage in primates. Our data support a putative trade-off between brain and body growth both within and across mammalian species [32,42]. In humans, developmental changes in the brain’s energy demands vary inversely with changes in growth rate between infancy and puberty, with the slowest pace of growth and fat deposition of the lifecycle coinciding with lifetime peak brain energy use in mid-childhood [1,43]. Similarly, the evolution of large brains has often been hypothesized to require reduced energy expenditures in other tissues [3,32, 44]. In support of this evolutionary trade-off, variation in the size of brains relative to body size across mammalian species correlates inversely with the deposition of adipose tissue [20]. Larger-brained primates have not only higher fasting blood glucose [6] but also lower adiposity [20]. The finding that both growth and adiposity are also reduced in mice inoculated with the GMs of high-EQ primates is consistent with the GM contributing to the partitioning of energy allocation between the brain and body and is broadly consistent with emerging evidence for a trade-off between brain energetics and both adiposity and growth rate across a wide taxonomic range [1,20, 45,47].

Importantly, human metabolism is known to be distinct from those of other primates in both juveniles and adults [7,48, 49], and this phenotype has been hypothesized to be an adaptation for supporting brains that are relatively larger even than those of other primates. For example, adult humans exhibit both increased glucose and adiposity [50]. While increased glucose production helps power the brain in real-time, increased adiposity serves as an important backup energy source for the brain in situations of reduced food availability and/or increased energetic demands [48]. This phenotype is likely linked to adult humans overall increased energy budget compared to other primates [7], which relaxes metabolic trade-offs and allows them to allocate more energy to all functions compared to other primates, including increased glucose production for their large brains and enhanced storage of glucose in the form of fat. Although our mice did not exhibit the increased adiposity typical of humans, we posit that this is the result of the constrained energy budgets of mice compared to humans. Since all mice in this study were from the same genetic background, they likely had similar, non-human energy budgets that necessitated the energetic trade-offs typical of primates even in mice inoculated with the human GM. It is also possible that our use of a single population of donor humans with low body mass index (BMI) influenced this result. However, human populations with extremely low adiposity still have higher adiposity than non-human primates [7].

**Fig. 4.**
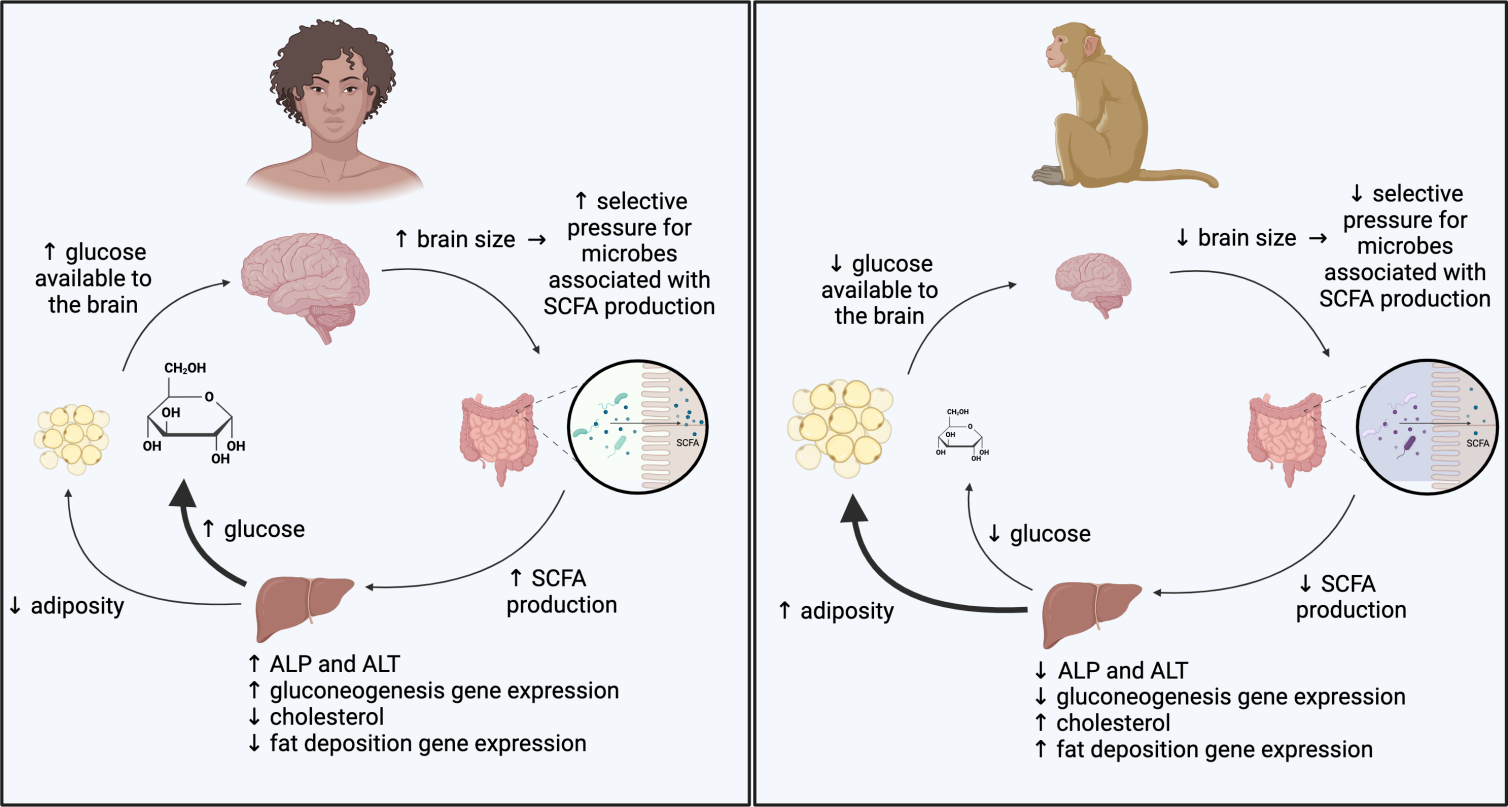
Putative model for microbial influences on the metabolism of high-EQ and low-EQ primates. Our findings indicate microbially mediated pathways through which the metabolism of high-EQ primates is biased towards energy production and the metabolism of low-EQ primates is biased towards energy storage.

Taken together, our data suggest that GM plays a causal role in differentially programming the metabolisms of different primate species in ways that are consistent with adult brain energetic requirements (Fig. 4). Specifically, the GMs of high-EQ primates produce increased concentrations of SCFAs, particularly acetate and propionate, which appear to promote both food consumption and gluconeogenesis and suppress lipogenesis in a mouse model. These host metabolic differences are associated with changes in liver gene expression, which may indicate a mechanism through which GM affects host metabolism. However, other mechanisms, such as alterations to energy and metabolite flux through key metabolic pathways, are also likely to be influential. Understanding dynamics in the small intestine, where nutrient absorption occurs and microbiome community composition is distinct, could also reveal other pathways of interest.

Some caveats exist for our study. For example, dissimilarities in collection conditions for human and non-human primate faecal samples may have introduced noise into our data. However, the direction of phenotypic effects in mice – those inoculated with human and squirrel monkey samples were similar to each other and distinct from those inoculated with macaque samples – reduces concerns of confounding bias. We also used a single population of each donor species, which could limit generalizability. We believe this constraint is unlikely given that previous studies have demonstrated that the effect of host species outweighs the effect of host environment/population across the order Primates [16], but future studies that incorporate multiple populations of donor species can further test this finding. Similarly, our study included two primate species with high EQ and one with low EQ. To further demonstrate that brain-related traits – and not other host species-specific traits – are driving the observed patterns, experiments with additional primate species donors are necessary.

The results of this study provide an important foundation upon which to continue investigations into the role of GM in host metabolic programming as it relates to the development, maintenance and evolution of the brain. While more encephalized primates require metabolic solutions for fuelling their brains during adulthood, exploring these host–microbe dynamics in juveniles undergoing active brain growth and/or with high energy costs related to processes such as synaptogenesis [1,51, 52] would provide further insight. Moving forward, additional research should systematically compare SCFA production patterns across a range of primate species and further target the specific mechanisms via which SCFAs alter host metabolism. It should also investigate the extent to which other microbially produced compounds are contributing to the observed patterns. For example, large amounts of serotonin are produced in the gut via interactions with GM [53], and serotonin can incite lipogenesis in the liver and suppress lipolysis in adipose tissues [54]. Similarly, microbe−liver interactions have been shown to mediate metabolic phenotypes via the production of trimethylamine *N*-oxide [55], which plays a role in brain development [56]. Knowledge of these pathways will provide important mechanistic insights into GM interactions with host metabolism and will also advance our understanding of the proximate factors driving brain growth and development in primates. Regardless of the mechanism, however, our finding that the GM helps shape host energy allocation patterns across host species suggests that microbes play an important role in scaffolding species differences in life histories, including the evolution of relatively large brains.

## Methods

### Stool sample collection

For human stool samples, adult participants of both sexes were recruited from the Evanston, IL, USA. Adults between 18 and 35 years of age with a BMI of 18.5–24.9 and no reported history of antibiotic use within the past 6 weeks collected samples at home using a commode collection device with a lid (Fisher Scientific 02-544-208). Participants stored samples in a cool location prior to delivering them to our laboratory within 24 h of collection. Samples were stored at −80 °C for several days until the collection was completed and were then processed by removing a 1 g piece of faecal material from the sample, placing it in a 15 ml tube and filling the tube with sterile 1× PBS containing 0.1% cysteine-HCl. All human samples were stored at −80 °C after arrival in the laboratory until use in the mouse experiment (<3 months). Participant recruitment and sampling procedures followed protocols approved by the Northwestern University Institutional Review Board (ID: STU00206091).

For macaques, stool samples were collected while individuals were either sedated or separated from their social groups. In the first case, individuals were anaesthetized with ketamine or telazol, and at least 100 g of faeces was collected from rhesus macaques by inserting a gloved finger into the rectum. In the latter case, voided faecal samples were collected in the catch pan of individuals that were temporarily singly housed. Adults from both sexes with a normal BMI and no antibiotic use in the previous 6 weeks were sampled. The samples were placed into pre-weighted tubes labelled with the animal ID, date and time of collection and collector’s initials. The samples were processed as described below and stored at −80 °C until experimental use (<1 month). The rhesus faecal samples were collected under M.D. Anderson IACUC #0000804-RN03, ‘Establishment and Maintenance of an SPF Rhesus Monkey Breeding and Research Program,’ and overlapping IACUC #00001437-RN02 ‘Preliminary characterization of chronic enterocolitis and early detection of colorectal cancer in rhesus macaques’.

For squirrel monkey stool samples, individual squirrel monkeys were removed from normal housing and placed into a clean housing unit until sufficient faecal samples were available, between 2 and 4 h. Faeces were collected without urine contamination from several spots on the housing floor using a pair of clean exam gloves and/or a wooden tongue depressor. Adults from both sexes with a normal BMI and no antibiotic use in the previous 6 weeks were sampled. The samples were placed into pre-weighted tubes labelled with the animal ID, date and time of collection and collector’s initials. The samples were processed as described below and stored at −80 °C until experimental use (<1 month). The squirrel monkey samples were collected under M.D. Anderson IACUC #00002148-RN00, ‘Characterization of the gut microbiome in squirrel monkeys’.

Squirrel monkey and macaque samples were processed by filling the sample tube with sterile 1× PBS containing 0.1% cysteine-HCl. The samples were vortexed at maximum speed for 5 min and allowed to settle for 5 min on ice. The upper liquid fraction (particle-free) was collected as the biome fraction. The aliquots of fraction were stored in glycerol (final concentration 10%) at −80 °C until shipment to Dr. Amato’s laboratory. They were stored at –80 °C in the Amato laboratory until use in the mouse experiment (6–12 months).

### Mouse inoculation and sample collection

Glycerol stocks from stool samples from five individuals of each species (including both sexes) were homogenized, pooled and diluted with sterile PBS to create a gavage solution. We chose which samples to pool pseudorandomly after checking for clear outliers in GM composition via 16S rRNA gene amplicon sequencing. Each solution was used to inoculate ten pair-housed weanling germ-free C57BL/6NTac mice via oral gavage. Gnotobiotic mice were maintained in a flexible-film isolator for the 60-day duration of the study, and treatments were run consecutively. The mice were fed an autoclavable standard chow diet (out of total calories: ~25% protein, ~17% fat, ~58% carbohydrates and ~5% fibre; Envigo Teklad LM-485), which they consumed *ad libitum*. Mice were housed in polysulfone shoebox cages with stainless-steel wire-bar lids on Alpha-dri^®^ bedding and Enviro-Dri^®^ nesting materials. Isolators were maintained at a temperature of 72 ± 2 °F, a relative humidity of 30–70%, and a 14:10 h light : dark cycle.

Mice were weighed 24 h after inoculation to establish a baseline weight, and faecal pellets and a 150 µl submandibular blood sample were collected. Subsequently, faecal and blood samples were collected weekly, and the mice were weighed weekly. Gavage was also repeated weekly to ensure the maintenance of the donor GM. Food consumption was estimated for each pair of animals every 14 days by measuring the quantity of food provided and the quantity of food not consumed over 24 h. At 30 and 60 days, a glucose tolerance test was administered by giving each mouse 2 g of glucose kg^−1^ of body weight after overnight fasting. Blood glucose levels were measured at five time points – 0, 15, 30, 60 and 120 minutes – using a tail prick and a handheld glucometer (Accu-Check). At 60 days, all mice were scanned using magnetic resonance imaging (MRI) to estimate adiposity at the Center for Advanced Molecular Imaging at Northwestern University. Subsequently, mice were euthanized with ketamine–xylazine. Organs were excised and weighed with a scale. The digestive tract was separated into sections (stomach, small intestine, caecum and large intestine). All digestive sections and the liver were frozen in a −80 °C freezer within 30 min of euthanasia. The Animal Care and Use Program at Northwestern University is accredited by AAALAC International, and all animal work was approved by the NU IACUC #IS00006555.

### MRI data generation

Each mouse was anaesthetized in an induction chamber using 3% isoflurane delivered in oxygen. After induction of anaesthesia, the mouse was positioned prone in a dedicated animal bed with isoflurane delivered through a nosecone at 1–2% as needed to maintain a respiratory rate of ~40 breaths per minute. Respiration and temperature were monitored with an SAI Model 1030 MR-compatible animal monitoring system (Small Animal Instruments Inc., Stony Brook, NY, USA) using a pillow-style pressure sensor and rectal temperature probe. The animal’s temperature was maintained using a warm water circulating blanket under the animal’s abdomen. Reference standards containing water and vegetable oil were positioned along the animal’s spine.

Imaging was performed on a 9.4T Bruker Biospec 9430 (Bruker Corporation, Billerica, MA, USA) with a 30 cm bore and 12 cm gradient insert, running Paravision 6.0.1. The radiofrequency coil was a 40 mm quadrature volume coil (Bruker) operating in transmit/receive mode. The mouse was imaged in two physical fields of view: first with the thorax centred in the coil, followed by ejection of the scanner bed, translation of the mouse along the *z*-axis to centre the abdomen in the coil and return of the scanner bed to the bore of the magnet. Fields of view covered anatomy from the base of the skull to the testes.

For each physical field of view, T_1_ weighted images were acquired using an accelerated spin echo sequence (T_1_ Rapid Acquisition with Relaxation Enhancement, T1-RARE) oriented axially. The following parameters were used: TR/TE = 1000 ms/6.25 ms, RARE factor 4, MTX = 256×256, FOV 4×4 cm, 15 slices, 1 mm slice thickness, 2 mm slice gap and 2 signal averages. Each acquisition time for each scan was ~1 min 15 s. Three interleaved slice packages were acquired, each shifted 1 mm relative to the previous, to achieve full slice coverage. For each slice geometry, one fat-suppressed scan and one otherwise identical non-fat-suppressed scan were acquired.

Images were exported from the scanner in DICOM format and imported into Amira 2019 software (Thermo Fisher Scientific, Waltham, MA, USA). Both the fat-suppressed and non-fat-suppressed images were normalized to the mean signal from the water tube. The torso and abdominal datasets were registered using the multi-planar alignment tool in Amira, cropped to reduce overlap and merged to create whole-body fat-suppressed and non-fat-suppressed datasets. A whole body ‘fat map’ was created by taking the difference of the two resultant datasets. A region of interest comprising the mouse body was created from the fat-suppressed image and overlaid on the fat map; fat voxels were identified as those with value >0.25 (mean ± 2 sd of the water tube values in the subtracted images). Fat percentage was calculated as the ratio of the volume of the fat region of interest to the total body volume.

### Intestinal pathology

At the end-point, a subset of caecum specimens from each species was excised, paraffin-embedded and processed for histopathological scoring. Haematoxylin and eosin-stained tissue sections were reviewed and scored by a board-certified pathologist in a blinded fashion. Scoring parameters included mucosal injury (crypt apoptosis, erosion and ulceration), lymphocytic inflammation (increased lymphocyte infiltrates into epithelia and in the lamina propria and thickening the subepithelial collagenous table), and neutrophile infiltration (neutrophils in the lamina propria, in the crypts and oedematous change).

### 16S rRNA gene sequencing and analysis

16S rRNA gene sequencing was used to assess GM composition from donor faecal samples, faecal samples collected from mice 4 days after the first inoculation with donor faecal samples and faecal samples collected from mice in the final week of the experiment. Established laboratory protocols were used to extract DNA and amplify the V4–V5 region of the 16S rRNA gene [57]. Briefly, DNA was extracted using a Qiagen DNeasy PowerSoil Kit with slight modifications to the manufacturer’s protocols. A two-step PCR amplification was performed using the 515 F-806R Earth Microbiome Project primers for the donor faecal samples and the 515 F-926R Earth Microbiome Project primers for the mouse faecal samples [58]. The resulting amplicons were cleaned using SequalPrep, followed by quantification with PicoGreen. Human, macaque and squirrel monkey donor samples were sequenced on the Illumina MiniSeq platform, and all mouse faecal samples were sequenced on the Illumina MiSeq 2×350 bp V3 platform. The bacterial genome copy number was estimated using real-time quantitative polymerase chain reaction (qPCR) of the *16S rRNA* gene on a ViiA 7 Real-Time PCR System (Thermo Fisher Scientific) [59]. Amplicon sequencing and qPCR were carried out by the Genome Research Core at the University of Illinois, Chicago.

Raw amplicon sequences were trimmed and quality-filtered, and ASVs were determined in QIIME2 v.2-2019.10 [60]. Taxonomy was assigned using the GreenGenes database [61]. We analysed single-end sequences for all samples (stocks, first time point and last time point), as well as analysed paired-end sequences for only the experimental samples (first and last time points). Negative controls were used for both DNA extraction and PCR. Negative controls had fewer sequences than actual samples in all cases and were, therefore, not included in further analyses. Prior to diversity analyses, the ASV table for all samples was rarefied to 10 177 sequences and the ASV table for only experimental samples was rarefied to 6573 sequences to retain all samples. Unweighted and weighted UniFrac distances, Faith’s phylogenetic diversity and Shannon diversity were calculated in QIIME2, and the q2-breakaway library within QIIME2 was used to calculate richness from unrarefied data [62]. To assess whether donor GMs were successfully established in the mice, pairwise distances between all donor species-time point combinations and core GM features for all samples from each donor species and all samples from each time point were calculated. Permutational analyses of variance (PERMANOVAs) were used to detect the effect of donor species identity and experimental time point (stock, first and last) on taxonomic composition with both unweighted and weighted UniFrac distances using the *vegan* package (v2.6.2) in R [63]. Generalized linear mixed-effects models (GLMMs) with a negative binomial distribution were used to examine the effect of donor species identity on the relative abundance of phyla, families and genera for unrarefied experimental samples and on the inferred absolute abundances of phyla, families and genera for the final time point only. GLMMs were run using the *glmmTMB* (v1.1.3) and *car* (v3.0.12) R packages [64,65], and family- and genus-level models were corrected for false discovery rate using the *fdrtool* (v1.2.16) R package [66]. R version 4.2.1 was used for all analyses.

### Metagenomic library preparation, sequencing and analysis

Libraries were constructed from DNA extracts from the final experimental time point (see above) using a NuGen Celero with Enzymatic Fragmentation kit using the manufacturer’s protocols. Libraries were pooled, size-selected to 400–600 bp using PippinPrep and sequenced on the Illumina NovaSeq SP 2×150 platform. Sequencing was performed at the Genome Research Core at the University of Illinois, Chicago.

Raw sequences were quality-filtered and trimmed using KneadData (http://huttenhower.sph.harvard.edu/kneaddata). HUMAnN2 processing pipeline was used for taxonomic and functional profiling [67]. The resulting gene family tables were regrouped into KEGG orthogroups, normalized to copies per million and split into stratified and unstratified tables. Pathway abundance tables were split into stratified and unstratified tables. Bray–Curtis and Jaccard distance matrices were calculated in QIIME v.2-2019.10 from unstratified gene family and pathway abundance tables. PERMANOVAs were used to detect the effect of donor species identity on gene family and pathway composition with both Bray–Curtis and Jaccard distances using the *vegan* package in R. Linear mixed-effects models were used to examine the effect of donor species identity on gene family and pathway abundance tables and were run using the *lme4* (v1.1–28) and *car* packages [68]. R version 4.2.1 was used for all analyses.

### SCFA metabolite extraction and analysis

SCFAs were extracted and derivatized from faecal samples collected from mice at the last time point. For all samples, one faecal pellet was used, and SCFA analyses were conducted on ice. Samples were first precisely weighed (most samples weighed 20–35 mg), homogenized with 50% acetonitrile (ACN) at a ratio of 1 g : 5 ml and vortexed for 5 min. Samples were then centrifuged at 4000***g*** at 4 °C for 10 min. SCFA derivatization included combining 40 µl of sample supernatant with 20 µl of 200 mM 3-nitrophenylhydrazine hydrochloride and 20 µl of 120 mM 1-ethyl-3-(3-dimethylaminopropyl)carbodiimide hydrochloride–6% pyridine solution and reacting samples at 40 °C for 30 min. Samples were diluted to 1 ml with 10% ACN to prevent sample loss from volatilization and stored at −80 °C until liquid chromatography-mass spectrometry (LC-MS/MS) analysis at the University of Illinois-Chicago Mass Spectrometry Core. Differences in the concentrations and relative proportions of acetate, butyrate, propionate and valerate across donor species groups were tested using a one-way ANOVA.

### RNA extraction, sequencing and analysis for liver samples

Mouse liver samples were micro-dissected using a tissue punch on the same lobe of the liver for every sample. Dissected mouse liver samples were weighed and homogenized in 250 µl lysis buffer with 0.7% beta-mercaptoethanol using a tissue homogenizer (Omni) for 15–20 s. Lysates were incubated at room temperature for 5 min and proceeded to RNA extraction on the KingFisher Flex (5400630 l Thermo Fisher Scientific) with an additional DNase step added according to the manufacturer’s protocols (Thermo Fisher Scientific, MagMax Total RNA isolation kit, A27828). RNA quality was determined using RNA 6000 Nano Assay with BioAnalyzer (Agilent) and RNA concentration was determined with Quant-it RNA High Sensitivity assay kit (Thermo Fisher Scientific, Q33140). RNA samples were normalized to 100 ng µl^−1^ and stored at −80 °C before submission for sequencing.

Extracted RNA samples were submitted to the Genome Sequence and Analysis Facility at the University of Texas at Austin for Tag-based RNA sequencing. This method is a cost-effective approach specifically designed to measure the abundances of polyadenylated transcripts yielding highly reliable data for differential gene expression analysis in well-annotated genomes [69]. Libraries were constructed with a protocol modified from Meyer *et al*. [70] and Lohman *et al*. [69] (updated version available Matz lab Github: https://github.com/z0on/tag-based_RNAseq/blob/master/TagSeq_sample_prep_june2019.docx). Reads were sequenced on the NovaSeq 6000 SR100 with minimum reads of 4 million and the target reads per sample of 5 million.

Raw reads were processed to obtain gene count data by following the TagSeq data processing pipeline provided based on Meyer *et al*. [70] and Lohman *et al*. [69] (maintained by Matz lab at UT Austin; https://github.com/z0on/tag-based_RNAseq/blob/master/tagSeq_processing_README.txt). Briefly, customized Perl script utilizing FASTX-Toolkit (http://hannonlab.cshl.edu/fastx_toolkit) and CUTADAPT v. 2.8 [71] was used to remove reads with a homo-polymer run of ‘*A*’ ≥ 8 bases and retain reads with minimum 20 bases and removal PCR duplicates. Processed reads were then mapped to the *Mus musculus* reference genome (Ensembl release 99) using Bowtie2 [72].

Differential gene expression analyses were conducted using DESeq2 in R v 3.6.2 [73]. The false discovery rate was controlled at 5% [74]. An adjusted *P*-value of 0.05 and a log twofold change >1.5 were used as cut-offs for differentially expressed genes.

For MiMeNet analysis, raw 16S rRNA counts were transformed using centred log-ratio transformation, and the union of differentially expressed genes across the comparisons between species was transformed using DESeq2’s variance stabilizing transformation. MiMeNet was then run using the transformed microbiome and the transformed gene expressions as inputs to obtain functional modules of microbes and genes (https://github.com/YDaiLab/MiMeNet). Gene set enrichment analysis was performed on each gene module identified by MiMeNet using the RITAN package and Reactome pathways [75]. MiMeNet was run using Python 3.7 and gene set enrichment was performed with R version 4.2.1.

For functional gene analysis, raw gene counts were filtered to remove any gene with fewer than ten counts across all samples. Differential genes were next identified using DESeq2 by comparing the three species in a pairwise fashion and using an adjusted *P*-value threshold of 0.05. Gene set enrichment analysis was performed on differential genes within each comparison with a fold-change value greater than 2 or less than −2 using the RITAN package and Reactome pathways. All analyses and plots were generated using R version 4.2.1.

### Physiological measurements and analysis

Blood concentrations of alkaline phosphatase (ALP), aspartate aminotransferase (AST), alanine aminotransferase (ALT), triglycerides, cholesterol, high-density lipoprotein, low-density lipoprotein and lipaemia were assessed by Idexx using standard assays. To quantify liver glycogen concentrations, liver tissue samples were dissected in a cryostat using a 2-mm biopsy punch (WP2020F, World Precision Instruments, Sarasota, FL) into 2-ml Reinforced Microvials (2007, BioSpec Products, Inc., Bartlesville, OK) containing 1 mm zirconia/silicon beads (11 079 110z, BioSpec). Dissected tissue samples were weighed [mean weight (sd) = 16.8 (3.5)], and then homogenized in 1 ml of assay buffer using the bead beater (607, BioSpec). The samples were boiled on a heat block at 100 °C for 10 min to inactivate enzyme activity and then centrifuged at 18 000 r.p.m. at 4 °C for 10 min. The supernatants were diluted 40-fold with assay buffer. Diluted homogenates were processed as described in the Glycogen Assay Kit manual (700 480, Cayman Chemical Company, Ann Arbor, MI). Differences in each physiological measure in response to donor species were assessed using a one-way ANOVA on either raw or transformed data, depending on the distribution.

## supplementary material

10.1099/mgen.0.001322 Uncited Supplementary Material 1.

## Abbreviations

ACN: acetonitrile
ALP: alkaline phosphatase
ALT: alanine aminotransferase
ASVs: amplicon sequence variants
BMI: body mass index
EQ: encephalization quotients
GLMMs: Generalized linear mixed effects models
GM: gut microbiota
RARE: rapid acquisition with relaxation enhancement
SCFAs: short chain fatty acids
