# Supplementary material for: The primate gut microbiota contributes to interspecific differences in host metabolism
Source: Microb Genom. 2024 Dec 2;10(12):001322. doi: 10.1099/mgen.0.001322 (PMC11893272; doi:10.1099/mgen.0.001322)
Supplement: Uncited Supplementary Material 1. [file mgen-10-01322-s001.pdf]

Supplementary Materials for

Title: The primate gut microbiota contributes to interspecific differences in host metabolism

Authors: Elizabeth K. Mallott[1,2]†, Sahana Kuthyar[1,3], Won Lee[4,5], Derek Reiman[6,7], Hongmei Jiang[8], Sriram Chitta[9], E. Alexandria Waters[10], Brian Layden[11], Ronen Sumagin[12], Laura D. Manzanares[12], Guan-Yu Yang[12], Maria Luisa Savo Sardaro[1], Stanton Gray[9], Lawrence E. Williams[9], Yang Dai[6], James P. Curley[4], Chad R. Haney[10], Emma R. Liechty[13], Christopher W. Kuzawa[1], Katherine R. Amato[1]*†

Corresponding author: katherine.amato@northwestern.edu

Supplemental Figures

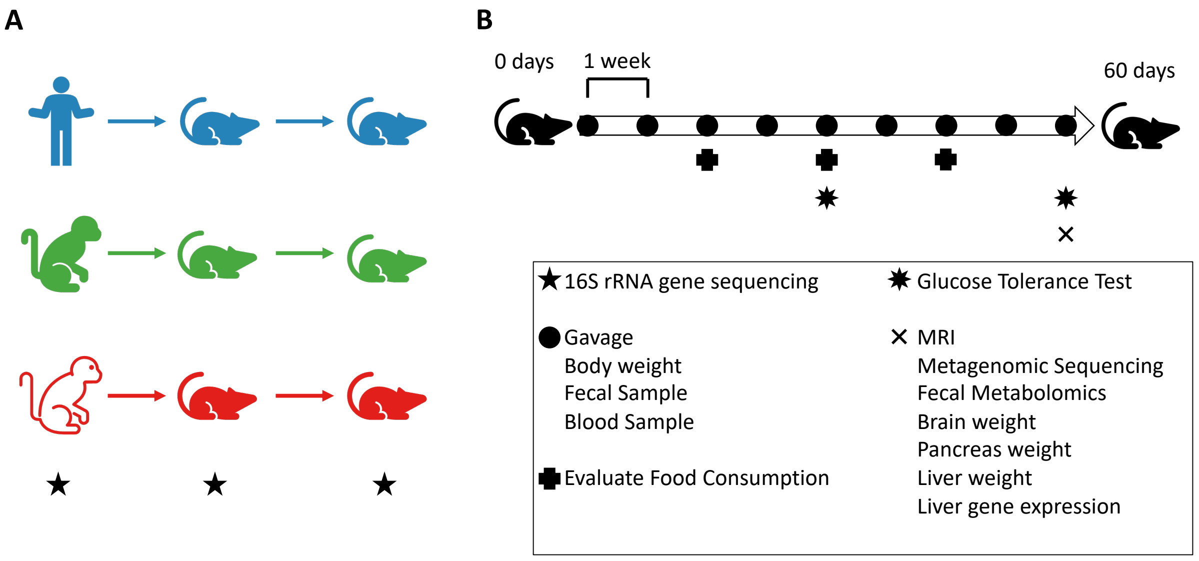

Fig. S1. Schematic of the experimental design used for mouse inoculations.

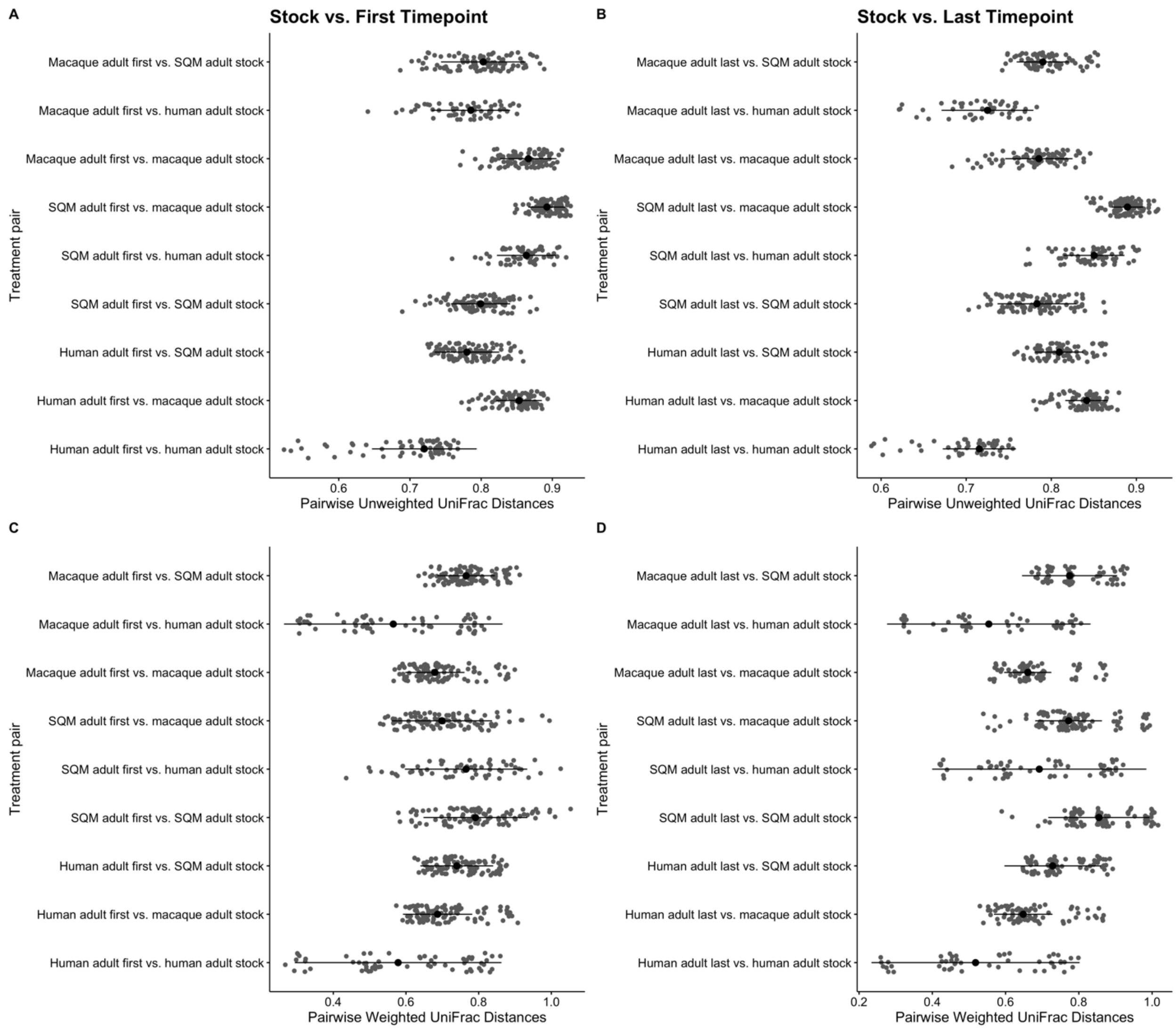

Figure S2. Pairwise distances unweighted (A and B) and weighted (C and D) between mice from each treatment group and donor stocks from the same species and stocks from different treatment groups. Panels A and C show distances from the first experimental timepoint to the stocks, while panels B and D show distances from the last experimental timepoint to the stocks. Centroids and error bars denote the median and interquartile range. 24 hours after the first oral gavage, the gut microbiome composition of mice from each donor group resembled the donor stock more than they resembled stocks from other donor groups. This pattern was maintained throughout the experiment so that at the final time point the gut microbiome composition of mice from each donor group resembled the donor stock more than they resembled stocks from other donor groups.

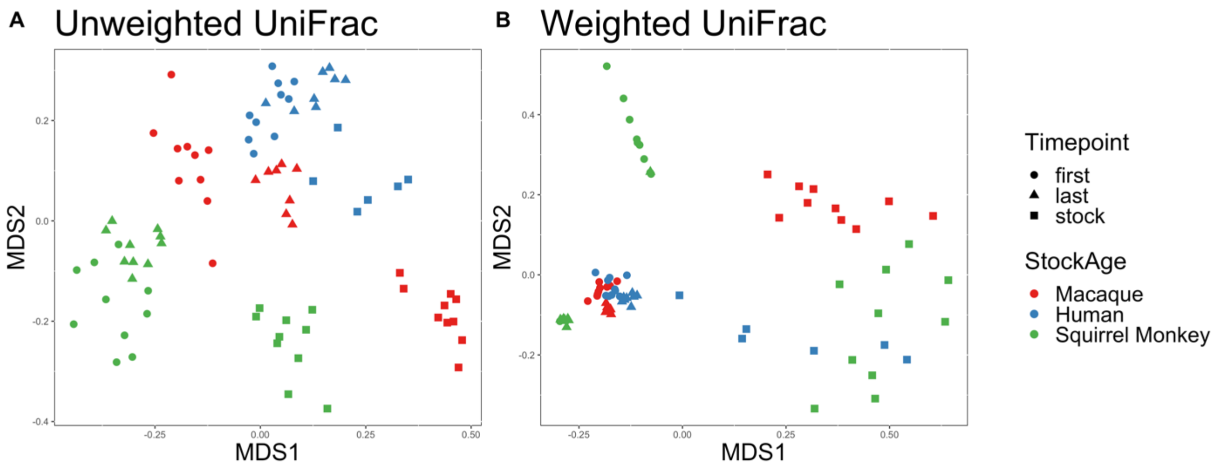

Figure S3. Unweighted (A) and weighted (B) UniFrac distances for donor stocks, first treatment timepoint, and last treatment timepoint for all species. Donor species significantly affected overall mouse microbiome taxonomic composition (unweighted UniFrac: $F_{2,95}$=23.5, R^2=0.3, p<0.001; weighted UniFrac: $F_{2,95}$=43.7, R^2=0.4, p<0.001), and the composition of donor stocks clustered with that of the fecal microbiome composition of mice inoculated with those stocks at both time points.

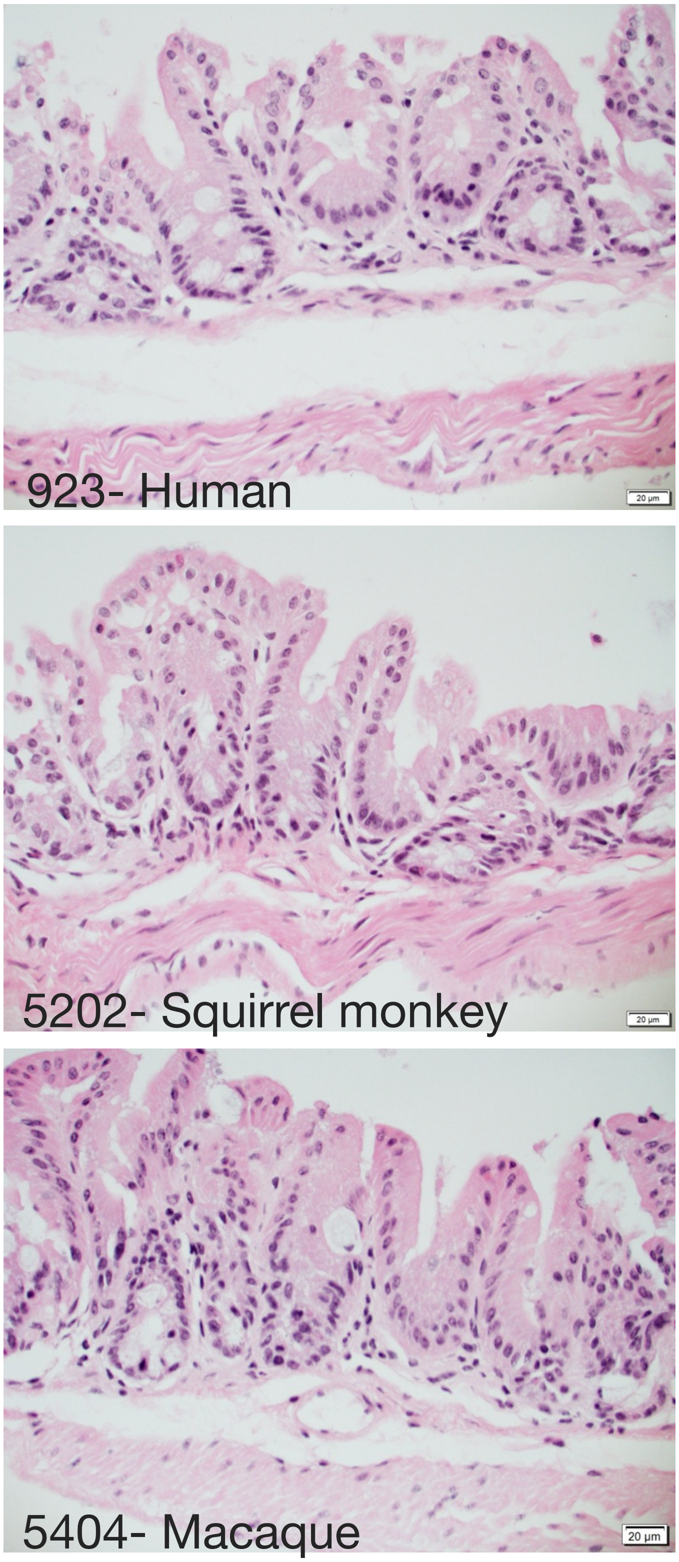

Figure S4. Representative examples of cecal tissue morphology from each treatment group.

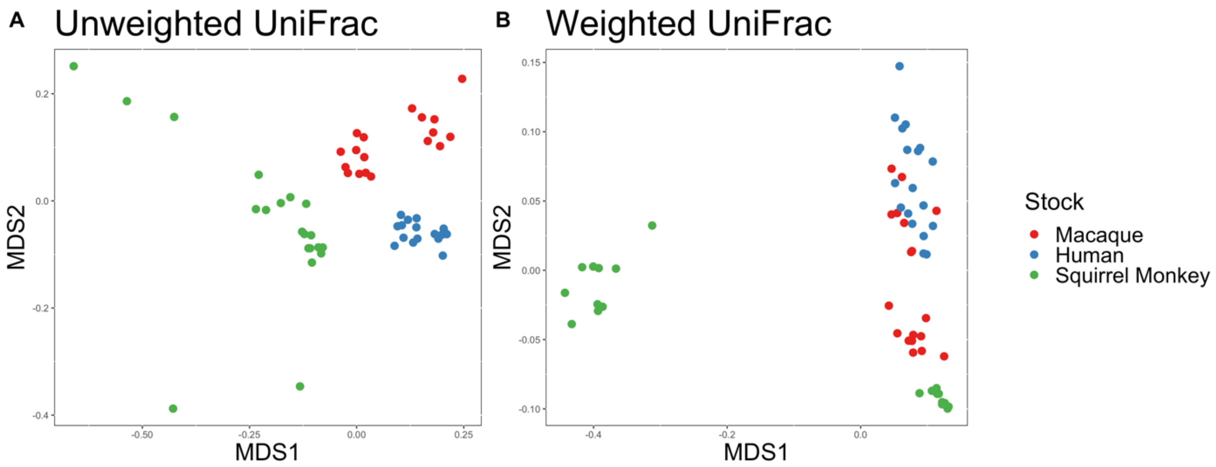

Figure S5. Unweighted (A) and weighted (B) UniFrac distances for last treatment timepoint for all species.

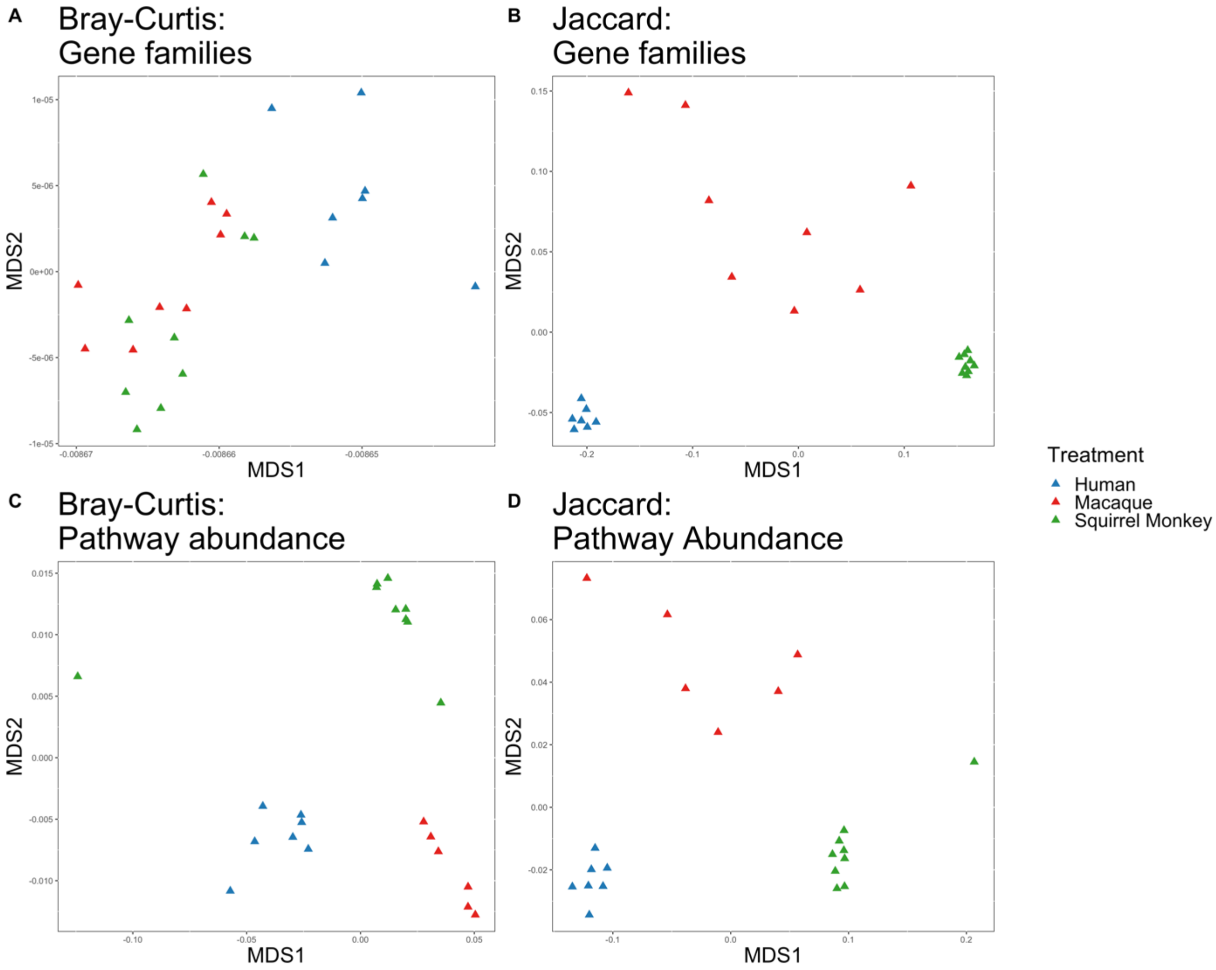

Figure S6. Bray-Curtis (A,C) and Jaccard (B, D) NMDS plots for gene families and pathway abundances calculated from metagenomic data. Donor species (Bray-Curtis, gene families: $F_{2,49}$=30.6, R^2=0.6, p<0.001; Jaccard, gene families: $F_{2,49}$=43.7, R^2=0.6, p<0.001; Bray-Curtis, pathway abundances: $F_{2,49}$=33.0, R^2=0.6, p<0.001; Jaccard, pathway abundances: $F_{2,49}$=37.5, R^2=0.6, p<0.001) influenced mouse gut microbiome function overall.

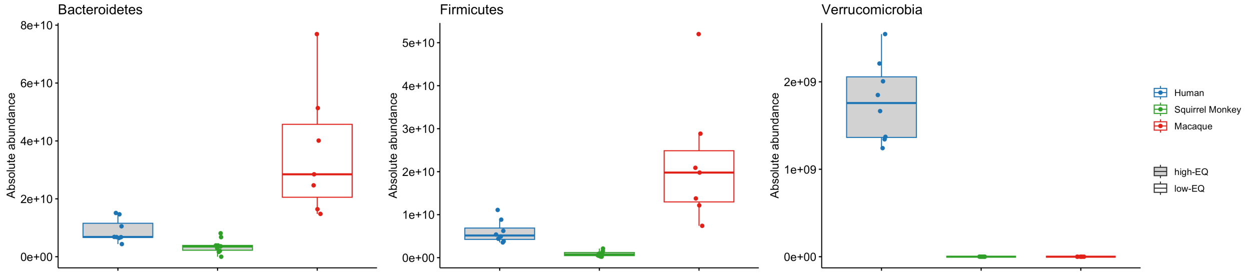

Figure S7. Inferred absolute abundances of bacterial phyla (calculated from 16S rRNA gene rt-qPCR results) that differed significantly between treatments during the last experimental timepoint. Donor species had significant influences on the absolute abundance of Bacteroidetes, Firmicutes, and Verrucomicrobia (all $p<0.05$).

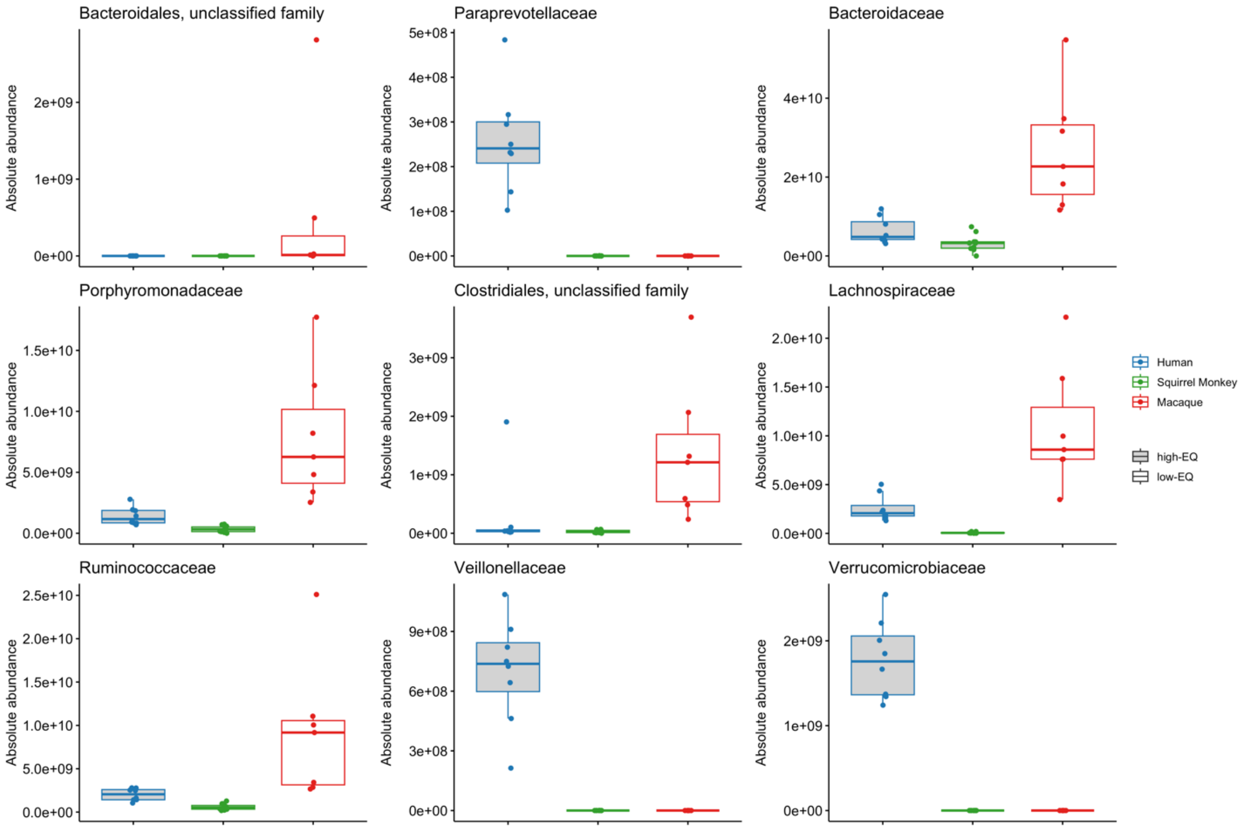

Figure S8. Inferred absolute abundances of bacterial families (calculated from 16S rRNA gene rt-qPCR results) that differed significantly between treatments during the last experimental timepoint. Donor species had a significant influence on the absolute abundance of Bacteroidaceae, Paraprevotellaceae, Porphyromonadaceae, Lachnospiraceae, Ruminococcaceae, Veillonellaceae, Verrucomicrobiaceae, an unclassified family of Bacteroidales, and an unclassified family of Clostridiales (all $q<0.05$).

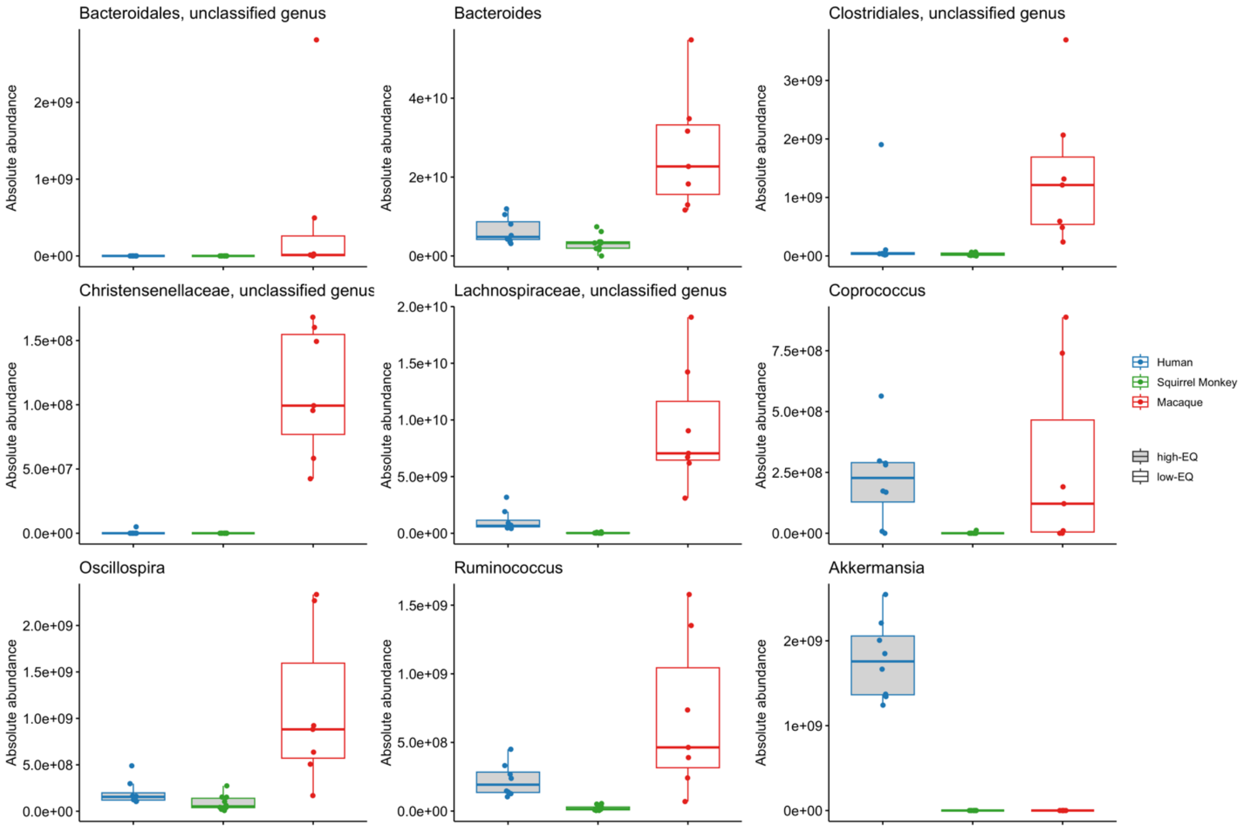

Figure S9. Inferred absolute abundances of bacterial genera (calculated from 16S rRNA gene rt-qPCR results) that differed significantly between treatments during the last experimental timepoint. Donor species had significant influences on the absolute abundance of *Bacteroides, Coprococcus, Oscillospira*, *Ruminococcus, Akkermansia,* and unclassified genera of Bacteroidales, Clostridiales, Christensenellaceae, and Lachnospiraceae (all $q<0.05$).

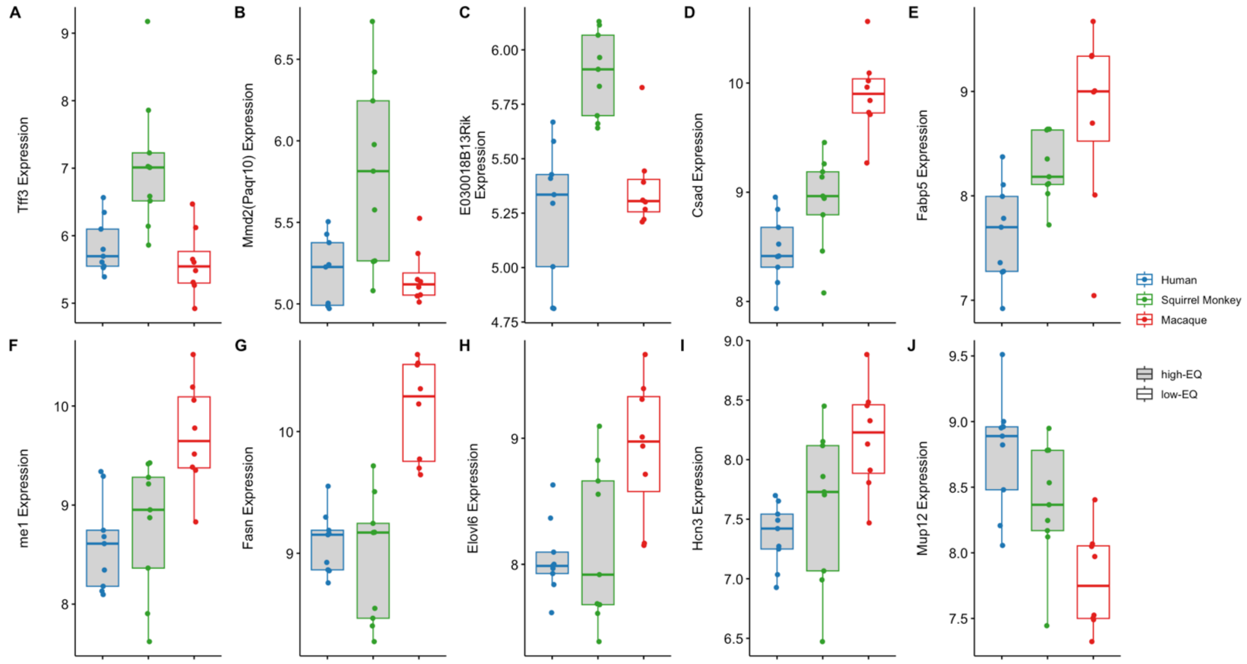

Figure S10. Genes that are differentially expressed in the liver (log2 fold change >1.5) between mice inoculated with human and squirrel monkey gut microbiomes (A-C), between mice inoculated with human and macaque gut microbiomes (D-I), and between mice inoculated with squirrel monkey and macaque gut microbiomes (J).

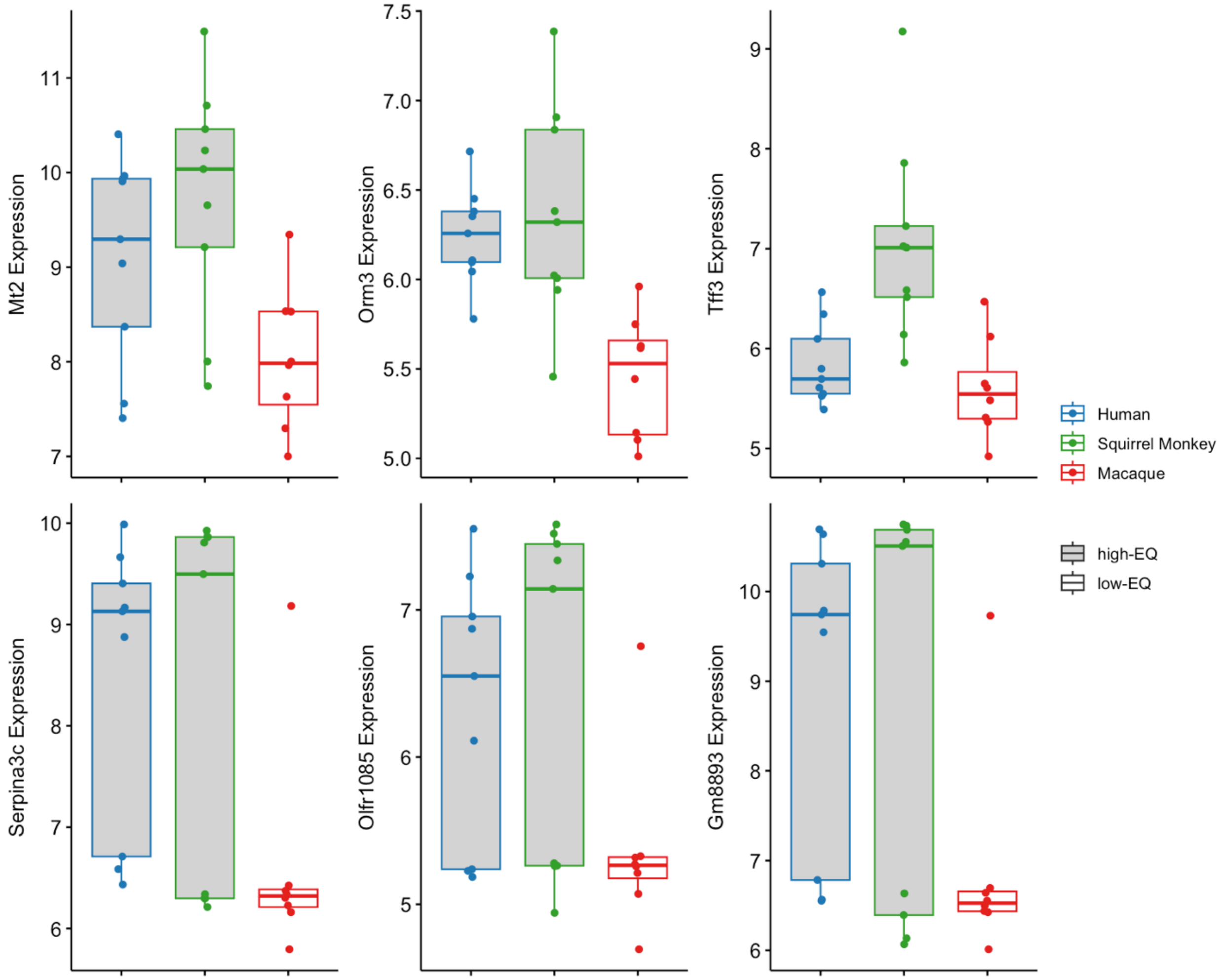

Figure S11. Genes that are differentially expressed in the liver (log2 fold change >1.5) between mice inoculated with low-EQ and high-EQ species' gut microbiomes.

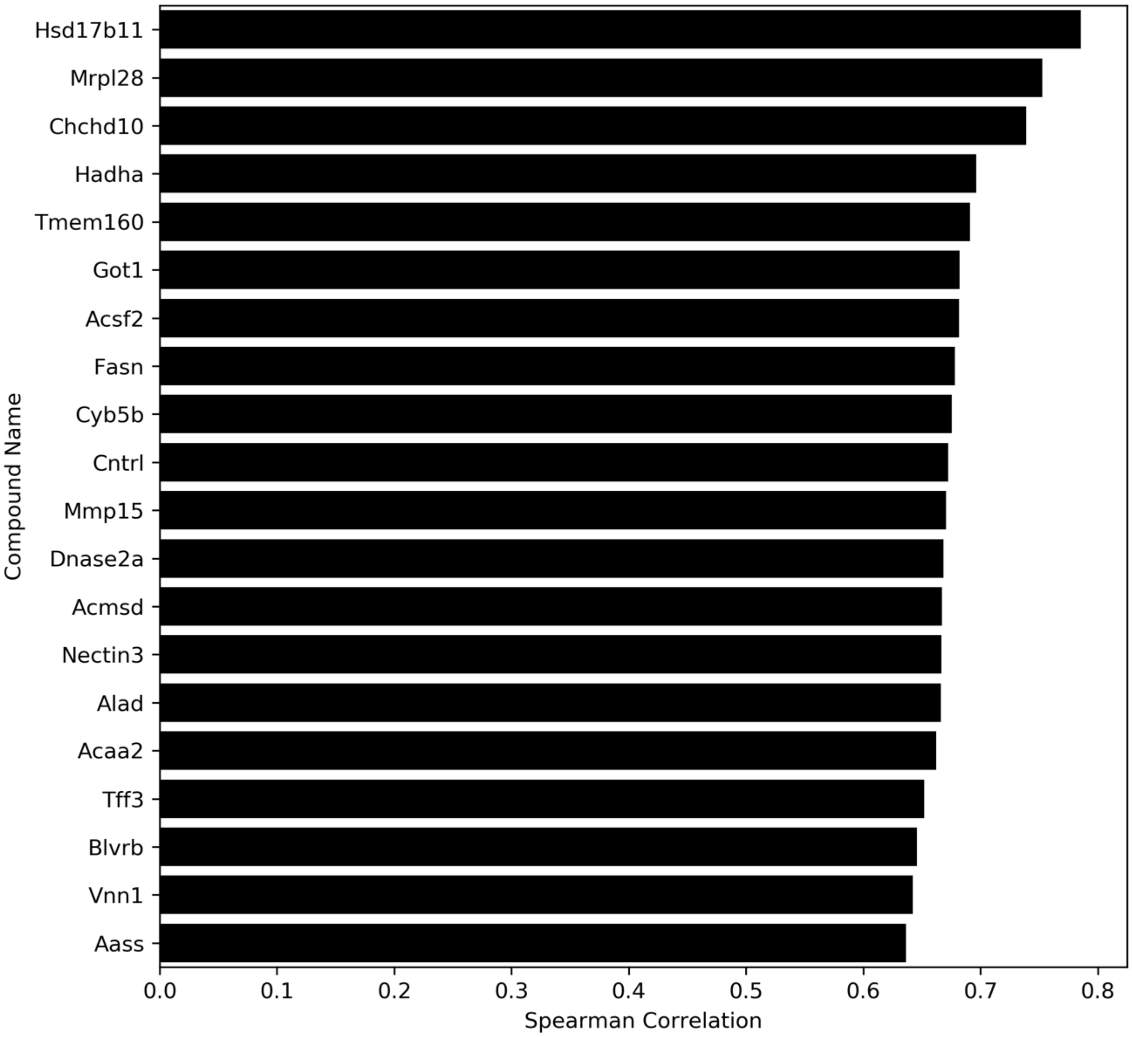

Figure S12. The top genes whose expression in mouse livers was predicted by 87 microbial ASVs according to MiMeNet analysis.

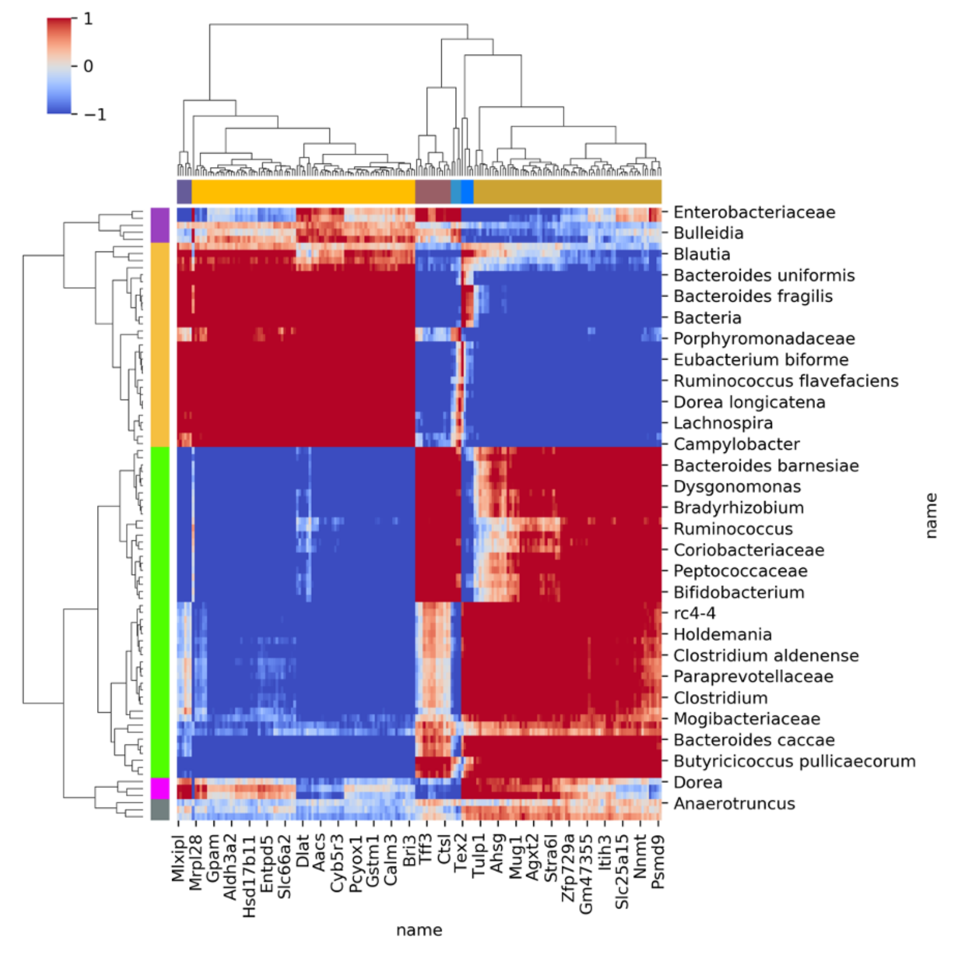

Figure S13. Genes predicted by microbial ASVs using MiMeNet were clustered into six different modules.

Supplemental Tables

Table S1. Microbial genera present in human donor stocks that were or were not detected in mouse fecal samples at the last experimental time point. Brackets represent suggested taxonomic changes, and missing taxonomic ranks indicate an unknown taxonomic assignment at that level.

Detected	Not detected
p__Actinobacteria;c__Actinobacteria;o__Bifidobacteriales;f__Bifidobacteriaceae;g__Bifidobacterium	k__Archaea;p__Euryarchaeota;c__Methanobacteria;o__Methanobacteriales;f__Methanobacteriaceae;g__Methanobrevibacter
p__Actinobacteria;c__Coriobacteriia;o__Coriobacteriales;f__Coriobacteriaceae;g__	p__Actinobacteria;c__Coriobacteriia;o__Coriobacteriales;f__Coriobacteriaceae;g__Adlercreutzia
p__Actinobacteria;c__Coriobacteriia;o__Coriobacteriales;f__Coriobacteriaceae;g__Collinsella	p__Actinobacteria;c__Coriobacteriia;o__Coriobacteriales;f__Coriobacteriaceae;g__Slackia
p__Bacteroidetes;__;__;__;__	p__Bacteroidetes;c__Bacteroidia;o__Bacteroidales;f__Porphyromonadaceae;g__Porphyromonas
p__Bacteroidetes;c__Bacteroidia;o__Bacteroidales;__;__	p__Bacteroidetes;c__Bacteroidia;o__Bacteroidales;f__S24-7;g__
p__Bacteroidetes;c__Bacteroidia;o__Bacteroidales;f__[Barnesiellaceae];__	p__Firmicutes;c__Bacilli;o__Turicibacterales;f__Turicibacteraceae;g__Turicibacter
p__Bacteroidetes;c__Bacteroidia;o__Bacteroidales;f__[Barnesiellaceae];g__	p__Firmicutes;c__Clostridia;o__Clostridiales;f__[Tissierellaceae];g__1-68
p__Bacteroidetes;c__Bacteroidia;o__Bacteroidales;f__[Odoribacteraceae];g__Butyricimonas	p__Firmicutes;c__Clostridia;o__Clostridiales;f__[Tissierellaceae];g__Peptoniphilus
p__Bacteroidetes;c__Bacteroidia;o__Bacteroidales;f__[Odoribacteraceae];g__Odoribacter	p__Firmicutes;c__Clostridia;o__Clostridiales;f__[Tissierellaceae];g__ph2
p__Bacteroidetes;c__Bacteroidia;o__Bacteroidales;f__[Paraprevotellaceae];g__	p__Firmicutes;c__Clostridia;o__Clostridiales;f__Clostridiaceae;g__Clostridium
p__Bacteroidetes;c__Bacteroidia;o__Bacteroidales;f__[Paraprevotellaceae];g__Paraprevotella	p__Firmicutes;c__Clostridia;o__Clostridiales;f__Clostridiaceae;g__SMB53
p__Bacteroidetes;c__Bacteroidia;o__Bacteroidales;f__Bacteroidaceae;g__Bacteroides	p__Firmicutes;c__Clostridia;o__Clostridiales;f__EtOH8;g__
p__Bacteroidetes;c__Bacteroidia;o__Bacteroidales;f__Porphyromonadaceae;g__Parabacteroides	p__Firmicutes;c__Clostridia;o__Clostridiales;f__Lachnospiraceae;g__Anaerostipes
p__Bacteroidetes;c__Bacteroidia;o__Bacteroidales;f__Prevotellaceae;g__Prevotella	p__Firmicutes;c__Clostridia;o__Clostridiales;f__Lachnospiraceae;g__Butyrivibrio
p__Bacteroidetes;c__Bacteroidia;o__Bacteroidales;f__Rikenellaceae;__	p__Firmicutes;c__Clostridia;o__Clostridiales;f__Lachnospiraceae;g__Lachnobacterium
p__Bacteroidetes;c__Bacteroidia;o__Bacteroidales;f__Rikenellaceae;g__	p__Firmicutes;c__Clostridia;o__Clostridiales;f__Lachnospiraceae;g__Lachnospira
p__Bacteroidetes;c__Bacteroidia;o__Bacteroidales;f__Rikenellaceae;g__Alistipes	p__Firmicutes;c__Clostridia;o__Clostridiales;f__Lachnospiraceae;g__Ruminococcus
p__Cyanobacteria;c__4C0d-2;o__YS2;f__;g__	p__Firmicutes;c__Clostridia;o__Clostridiales;f__Veillonellaceae;g__Acidaminococcus
p__Firmicutes;__;__;__;__	p__Firmicutes;c__Clostridia;o__Clostridiales;f__Veillonellaceae;g__Dialister

p__Firmicutes;c__Bacilli;o__Lactobacillales;f__Streptococcaceae;g__Streptococcus

p__Firmicutes;c__Clostridia;__;__;__

p__Firmicutes;c__Clostridia;o__Clostridiales;__;__

p__Firmicutes;c__Clostridia;o__Clostridiales;f__[Mogibacteriaceae];__

p__Firmicutes;c__Clostridia;o__Clostridiales;f__[Mogibacteriaceae];g__

p__Firmicutes;c__Clostridia;o__Clostridiales;f__Christensenellaceae;g__

p__Firmicutes;c__Clostridia;o__Clostridiales;f__Christensenellaceae;g__Christensenella

p__Firmicutes;c__Clostridia;o__Clostridiales;f__Lachnospiraceae;__

p__Firmicutes;c__Clostridia;o__Clostridiales;f__Lachnospiraceae;g__[Ruminococcus]

p__Firmicutes;c__Clostridia;o__Clostridiales;f__Lachnospiraceae;g__Blautia

p__Firmicutes;c__Clostridia;o__Clostridiales;f__Lachnospiraceae;g__Clostridium

p__Firmicutes;c__Clostridia;o__Clostridiales;f__Lachnospiraceae;g__Coprococcus

p__Firmicutes;c__Clostridia;o__Clostridiales;f__Lachnospiraceae;g__Dorea

p__Firmicutes;c__Clostridia;o__Clostridiales;f__Lachnospiraceae;g__Roseburia

p__Firmicutes;c__Clostridia;o__Clostridiales;f__Veillonellaceae;g__Megasphaera

p__Firmicutes;c__Clostridia;o__Clostridiales;f__Veillonellaceae;g__Mitsuokella

p__Firmicutes;c__Clostridia;o__Clostridiales;f__Veillonellaceae;g__Veillonella

p__Firmicutes;c__Erysipelotrichi;o__Erysipelotrichales;f__Erysipelotrichaceae;__

p__Firmicutes;c__Erysipelotrichi;o__Erysipelotrichales;f__Erysipelotrichaceae;g__

p__Firmicutes;c__Erysipelotrichi;o__Erysipelotrichales;f__Erysipelotrichaceae;g__RFN20

p__Lentisphaerae;c__[Lentisphaeria];o__Victivallales;f__Victivallaceae;g__

p__Lentisphaerae;c__[Lentisphaeria];o__Victivallales;f__Victivallaceae;g__Victivallis

p__Proteobacteria;c__Alphaproteobacteria;__;__;__

p__Proteobacteria;c__Betaproteobacteria;o__Burkholderiales;f__Comamonadaceae;__

p__Proteobacteria;c__Epsilonproteobacteria;o__Campylobacterales;f__Campylobacteraceae;g__Campylobacter

p__Proteobacteria;c__Gammaproteobacteria;o__Pasteurellales;f__Pasteurellaceae;g__Haemophilus

p__Tenericutes;c__Mollicutes;o__Anaeroplasmatales;f__Anaeroplasmataceae;g__

p__Tenericutes;c__RF3;o__ML615J-28;f__;g__

p__Firmicutes;c__Clostridia;o__Clostridiales;f__Peptococcaceae;g__rc4-4

p__Firmicutes;c__Clostridia;o__Clostridiales;f__Peptostreptococcaceae;__

p__Firmicutes;c__Clostridia;o__Clostridiales;f__Ruminococcaceae;__

p__Firmicutes;c__Clostridia;o__Clostridiales;f__Ruminococcaceae;g__

p__Firmicutes;c__Clostridia;o__Clostridiales;f__Ruminococcaceae;g__Anaerotruncus

p__Firmicutes;c__Clostridia;o__Clostridiales;f__Ruminococcaceae;g__Butyricicoccus

p__Firmicutes;c__Clostridia;o__Clostridiales;f__Ruminococcaceae;g__Clostridium

p__Firmicutes;c__Clostridia;o__Clostridiales;f__Ruminococcaceae;g__Faecalibacterium

p__Firmicutes;c__Clostridia;o__Clostridiales;f__Ruminococcaceae;g__Gemmiger

p__Firmicutes;c__Clostridia;o__Clostridiales;f__Ruminococcaceae;g__Oscillospira

p__Firmicutes;c__Clostridia;o__Clostridiales;f__Ruminococcaceae;g__Ruminococcus

p__Firmicutes;c__Clostridia;o__Clostridiales;f__Veillonellaceae;g__Phascolarctobacterium

p__Firmicutes;c__Erysipelotrichi;o__Erysipelotrichales;f__Erysipelotrichaceae;g__[Eubacterium]

p__Firmicutes;c__Erysipelotrichi;o__Erysipelotrichales;f__Erysipelotrichaceae;g__Clostridium

p__Firmicutes;c__Erysipelotrichi;o__Erysipelotrichales;f__Erysipelotrichaceae;g__Holdemania

p__Proteobacteria;__;__;__;__

p__Proteobacteria;c__Alphaproteobacteria;o__RF32;f__;g__

p__Proteobacteria;c__Betaproteobacteria;o__Burkholderiales;__;__
p__Proteobacteria;c__Betaproteobacteria;o__Burkholderiales;f__Alcaligenaceae;g__Sutterella
p__Proteobacteria;c__Betaproteobacteria;o__Burkholderiales;f__Oxalobacteraceae;g__Oxalobacter
p__Proteobacteria;c__Deltaproteobacteria;o__Desulfovibrionales;f__Desulfovibrionaceae;g__Bilophila
p__Proteobacteria;c__Deltaproteobacteria;o__Desulfovibrionales;f__Desulfovibrionaceae;g__Desulfovibrio
p__Proteobacteria;c__Gammaproteobacteria;o__Enterobacteriales;f__Enterobacteriaceae;__
p__Tenericutes;c__Mollicutes;o__RF39;f__;g__
p__Verrucomicrobia;c__Verrucomicrobiae;o__Verrucomicrobiales;f__Verrucomicrobiaceae;g__Akkermansia

Table S2. Microbial genera present in macaque donor stocks that were or were not detected in mouse fecal samples at the last experimental time point. Brackets represent suggested taxonomic changes, and missing taxonomic ranks indicate an unknown taxonomic assignment at that level.

Detected	Not detected
k__Archaea;p__Euryarchaeota;c__Methanobacteria;o__Methanobacteriales;f__Methanobacteriaceae;g__Methanobrevibacter	k__Archaea;p__Euryarchaeota;c__Methanobacteria;o__Methanobacteriales;f__Methanobacteriaceae;g__Methanosphaera
p__Actinobacteria;c__Coriobacteriia;o__Coriobacteriales;f__Coriobacteriaceae;__	k__Archaea;p__Euryarchaeota;c__Thermoplasmata;o__E2;f__[Methanomassiliicoccaceae];g__vadinCA11
p__Actinobacteria;c__Coriobacteriia;o__Coriobacteriales;f__Coriobacteriaceae;g__	p__Actinobacteria;c__Coriobacteriia;o__Coriobacteriales;f__Coriobacteriaceae;g__Olsenella
p__Actinobacteria;c__Coriobacteriia;o__Coriobacteriales;f__Coriobacteriaceae;g__Collinsella	p__Actinobacteria;c__Coriobacteriia;o__Coriobacteriales;f__Coriobacteriaceae;g__Slackia
p__Bacteroidetes;__;__;__;__	p__Bacteroidetes;c__Bacteroidia;o__Bacteroidales;f__;g__
p__Bacteroidetes;c__Bacteroidia;o__Bacteroidales;__;__	p__Bacteroidetes;c__Bacteroidia;o__Bacteroidales;f__[Paraprevotellaceae];g__
p__Bacteroidetes;c__Bacteroidia;o__Bacteroidales;f__[Odoribacteraceae];g__Butyricimonas	p__Bacteroidetes;c__Bacteroidia;o__Bacteroidales;f__[Paraprevotellaceae];g__[Prevotella]
p__Bacteroidetes;c__Bacteroidia;o__Bacteroidales;f__[Odoribacteraceae];g__Odoribacter	p__Bacteroidetes;c__Bacteroidia;o__Bacteroidales;f__[Paraprevotellaceae];g__CF231
p__Bacteroidetes;c__Bacteroidia;o__Bacteroidales;f__[Paraprevotellaceae];g__YRC22	p__Bacteroidetes;c__Bacteroidia;o__Bacteroidales;f__Bacteroidaceae;g__Bacteroides
p__Bacteroidetes;c__Bacteroidia;o__Bacteroidales;f__Porphyromonadaceae;g__Parabacteroides	p__Bacteroidetes;c__Bacteroidia;o__Bacteroidales;f__p-2534-18B5;g__
p__Bacteroidetes;c__Bacteroidia;o__Bacteroidales;f__Prevotellaceae;g__Prevotella	p__Bacteroidetes;c__Bacteroidia;o__Bacteroidales;f__Porphyromonadaceae;__
p__Cyanobacteria;c__4C0d-2;o__YS2;f__;g__	p__Bacteroidetes;c__Bacteroidia;o__Bacteroidales;f__Prevotellaceae;g__
p__Firmicutes;c__Bacilli;o__Lactobacillales;f__Lactobacillaceae;g__Lactobacillus	p__Bacteroidetes;c__Bacteroidia;o__Bacteroidales;f__RF16;g__
p__Firmicutes;c__Clostridia;__;__;__	p__Bacteroidetes;c__Bacteroidia;o__Bacteroidales;f__Rikenellaceae;g__
p__Firmicutes;c__Clostridia;o__Clostridiales;__;__	p__Bacteroidetes;c__Bacteroidia;o__Bacteroidales;f__S24-7;g__
p__Firmicutes;c__Clostridia;o__Clostridiales;f__[Mogibacteriaceae];__	p__Elusimicrobia;c__Elusimicrobia;o__Elusimicrobiales;f__Elusimicrobiaceae;g__
p__Firmicutes;c__Clostridia;o__Clostridiales;f__[Mogibacteriaceae];g__	p__Elusimicrobia;c__Elusimicrobia;o__Elusimicrobiales;f__Elusimicrobiaceae;g__Elusimicrobium
p__Firmicutes;c__Clostridia;o__Clostridiales;f__Lachnospiraceae;g__[Ruminococcus]	p__Fibrobacteres;c__Fibrobacteria;o__Fibrobacterales;f__Fibrobacteraceae;g__Fibrobacter
p__Firmicutes;c__Clostridia;o__Clostridiales;f__Lachnospiraceae;g__Anaerostipes	p__Firmicutes;__;__;__;__
p__Firmicutes;c__Clostridia;o__Clostridiales;f__Lachnospiraceae;g__Blautia	p__Firmicutes;c__Bacilli;o__Lactobacillales;f__Lactobacillaceae;__
p__Firmicutes;c__Clostridia;o__Clostridiales;f__Lachnospiraceae;g__Butyrivibrio	p__Firmicutes;c__Bacilli;o__Lactobacillales;f__Streptococcaceae;g__Streptococcus

p__Firmicutes;c__Clostridia;o__Clostridiales;f__Lachnospiraceae;g__Clostridium
p__Firmicutes;c__Clostridia;o__Clostridiales;f__Lachnospiraceae;g__Coprococcus
p__Firmicutes;c__Clostridia;o__Clostridiales;f__Lachnospiraceae;g__Dorea
p__Firmicutes;c__Clostridia;o__Clostridiales;f__Lachnospiraceae;g__Lachnospira
p__Firmicutes;c__Clostridia;o__Clostridiales;f__Lachnospiraceae;g__Oribacterium
p__Firmicutes;c__Clostridia;o__Clostridiales;f__Lachnospiraceae;g__Pseudobutyrivibrio
p__Firmicutes;c__Clostridia;o__Clostridiales;f__Lachnospiraceae;g__Roseburia
p__Firmicutes;c__Clostridia;o__Clostridiales;f__Peptostreptococcaceae;__
p__Firmicutes;c__Clostridia;o__Clostridiales;f__Ruminococcaceae;__
p__Firmicutes;c__Clostridia;o__Clostridiales;f__Ruminococcaceae;g__
p__Firmicutes;c__Clostridia;o__Clostridiales;f__Ruminococcaceae;g__Butyricicoccus
p__Firmicutes;c__Clostridia;o__Clostridiales;f__Ruminococcaceae;g__Faecalibacterium
p__Firmicutes;c__Clostridia;o__Clostridiales;f__Ruminococcaceae;g__Gemmiger
p__Firmicutes;c__Clostridia;o__Clostridiales;f__Ruminococcaceae;g__Oscillospira
p__Firmicutes;c__Clostridia;o__Clostridiales;f__Ruminococcaceae;g__Ruminococcus
p__Firmicutes;c__Erysipelotrichi;o__Erysipelotrichales;f__Erysipelotrichaceae;g__[Eubacterium]
p__Firmicutes;c__Erysipelotrichi;o__Erysipelotrichales;f__Erysipelotrichaceae;g__Bulleidia
p__Firmicutes;c__Erysipelotrichi;o__Erysipelotrichales;f__Erysipelotrichaceae;g__p-75-a5
p__Proteobacteria;c__Alphaproteobacteria;o__RF32;f__;g__
p__Proteobacteria;c__Betaproteobacteria;o__Burkholderiales;__;__
p__Proteobacteria;c__Deltaproteobacteria;o__Desulfovibrionales;f__Desulfovibrionaceae;g__
p__Proteobacteria;c__Epsilonproteobacteria;o__Campylobacterales;f__Campylobacteraceae;g__Campylobacter
p__Proteobacteria;c__Gammaproteobacteria;o__Aeromonadales;f__Succinivibrionaceae;g__Succinivibrio
p__Proteobacteria;c__Gammaproteobacteria;o__Enterobacteriales;f__Enterobacteriaceae;__
p__Firmicutes;c__Clostridia;o__Clostridiales;f__[Mogibacteriaceae];g__Mogibacterium
p__Firmicutes;c__Clostridia;o__Clostridiales;f__Christensenellaceae;g__
p__Firmicutes;c__Clostridia;o__Clostridiales;f__Clostridiaceae;__
p__Firmicutes;c__Clostridia;o__Clostridiales;f__Clostridiaceae;g__Clostridium
p__Firmicutes;c__Clostridia;o__Clostridiales;f__Clostridiaceae;g__Sarcina
p__Firmicutes;c__Clostridia;o__Clostridiales;f__Clostridiaceae;g__SMB53
p__Firmicutes;c__Clostridia;o__Clostridiales;f__Dehalobacteriaceae;g__Dehalobacterium
p__Firmicutes;c__Clostridia;o__Clostridiales;f__Lachnospiraceae;__
p__Firmicutes;c__Clostridia;o__Clostridiales;f__Lachnospiraceae;g__Shuttleworthia
p__Firmicutes;c__Clostridia;o__Clostridiales;f__Peptococcaceae;g__Peptococcus
p__Firmicutes;c__Clostridia;o__Clostridiales;f__Ruminococcaceae;g__Clostridium
p__Firmicutes;c__Clostridia;o__Clostridiales;f__Veillonellaceae;__
p__Firmicutes;c__Clostridia;o__Clostridiales;f__Veillonellaceae;g__
p__Firmicutes;c__Clostridia;o__Clostridiales;f__Veillonellaceae;g__Acidaminococcus
p__Firmicutes;c__Clostridia;o__Clostridiales;f__Veillonellaceae;g__Anaerovibrio
p__Firmicutes;c__Clostridia;o__Clostridiales;f__Veillonellaceae;g__Dialister
p__Firmicutes;c__Clostridia;o__Clostridiales;f__Veillonellaceae;g__Megasphaera
p__Firmicutes;c__Clostridia;o__Clostridiales;f__Veillonellaceae;g__Phascolarctobacterium
p__Firmicutes;c__Clostridia;o__Clostridiales;f__Veillonellaceae;g__Veillonella
p__Firmicutes;c__Erysipelotrichi;o__Erysipelotrichales;f__Erysipelotrichaceae;__
p__Firmicutes;c__Erysipelotrichi;o__Erysipelotrichales;f__Erysipelotrichaceae;g__
p__Firmicutes;c__Erysipelotrichi;o__Erysipelotrichales;f__Erysipelotrichaceae;g__Asteroleplasma
p__Firmicutes;c__Erysipelotrichi;o__Erysipelotrichales;f__Erysipelotrichaceae;g__Catenibacterium
p__Firmicutes;c__Erysipelotrichi;o__Erysipelotrichales;f__Erysipelotrichaceae;g__Coprobacillus

p__Spirochaetes;c__Spirochaetes;o__Sphaerochaetales;f__Sphaerochaetaceae;g__Sphaerochaeta	p__Firmicutes;c__Erysipelotrichi;o__Erysipelotrichales;f__Erysipelotrichaceae;g__RFN20
p__Tenericutes;c__Mollicutes;o__RF39;f__;g__	p__Fusobacteria;c__Fusobacteriia;o__Fusobacteriales;f__Fusobacteriaceae;__
	p__Fusobacteria;c__Fusobacteriia;o__Fusobacteriales;f__Fusobacteriaceae;g__Cetobacterium
	p__Lentisphaerae;c__[Lentisphaeria];o__Victivallales;f__Victivallaceae;g__
	p__Lentisphaerae;c__[Lentisphaeria];o__Victivallales;f__Victivallaceae;g__Victivallis
	p__Lentisphaerae;c__[Lentisphaeria];o__Z20;f__R4-45B;g__
	p__Planctomycetes;__;__;__;__
	p__Proteobacteria;__;__;__;__
	p__Proteobacteria;c__Alphaproteobacteria;__;__;__
	p__Proteobacteria;c__Betaproteobacteria;o__Burkholderiales;f__Comamonadaceae;__
	p__Proteobacteria;c__Deltaproteobacteria;__;__;__
	p__Proteobacteria;c__Deltaproteobacteria;o__Desulfovibrionales;f__Desulfovibrionaceae;__
	p__Proteobacteria;c__Deltaproteobacteria;o__Desulfovibrionales;f__Desulfovibrionaceae;g__Desulfovibrio
	p__Proteobacteria;c__Epsilonproteobacteria;o__Campylobacterales;f__Helicobacteraceae;__
	p__Proteobacteria;c__Epsilonproteobacteria;o__Campylobacterales;f__Helicobacteraceae;g__Flexispira
	p__Proteobacteria;c__Epsilonproteobacteria;o__Campylobacterales;f__Helicobacteraceae;g__Helicobacter
	p__Proteobacteria;c__Gammaproteobacteria;__;__;__
	p__Proteobacteria;c__Gammaproteobacteria;o__Aeromonadales;f__Succinivibrionaceae;g__Anaerobiospirillum
	p__Proteobacteria;c__Gammaproteobacteria;o__Pasteurellales;f__Pasteurellaceae;g__Actinobacillus
	p__Proteobacteria;c__Gammaproteobacteria;o__Pasteurellales;f__Pasteurellaceae;g__Aggregatibacter
	p__Proteobacteria;c__Gammaproteobacteria;o__Pseudomonadales;f__Pseudomonadaceae;g__Pseudomonas
	p__Spirochaetes;c__[Brachyspirae];o__[Brachyspirales];f__Brachyspiraceae;g__Brachyspira
	p__Spirochaetes;c__Spirochaetes;__;__;__

	p__Spirochaetes;c__Spirochaetes;o__Spirochaetales;f__Spirochaetaceae;g__Treponema
	p__Tenericutes;__;__;__;__
	p__Tenericutes;c__Mollicutes;__;__;__
	p__Tenericutes;c__Mollicutes;o__Anaeroplasmatales;f__Anaeroplasmataceae;g__
	p__Tenericutes;c__Mollicutes;o__Anaeroplasmatales;f__Anaeroplasmataceae;g__Anaeroplasma
	p__Tenericutes;c__RF3;o__ML615J-28;f__;g__
	p__TM7;c__TM7-3;o__CW040;f__F16;g__
	p__Verrucomicrobia;c__Opitutae;o__[Cerasicoccales];f__[Cerasicoccaceae];g__
	p__Verrucomicrobia;c__Verruco-5;o__WCHB1-41;__;__
	p__Verrucomicrobia;c__Verruco-5;o__WCHB1-41;f__RFP12;g__
	p__WPS-2;c__;o__;f__;g__

Table S3. Microbial genera present in squirrel monkey donor stocks that were or were not detected in mouse fecal samples at the last experimental time point. Brackets represent suggested taxonomic changes, and missing taxonomic ranks indicate an unknown taxonomic assignment at that level.

Detected	Not detected
p__Actinobacteria;c__Actinobacteria;o__Bifidobacteriales;f__Bifidobacteriaceae;g__Bifidobacterium	p__Actinobacteria;c__Actinobacteria;o__Actinomycetales;f__Actinomycetaceae;g__Actinomyces
p__Actinobacteria;c__Coriobacteriia;o__Coriobacteriales;f__Coriobacteriaceae;__	p__Actinobacteria;c__Actinobacteria;o__Actinomycetales;f__Corynebacteriaceae;g__Corynebacterium
p__Actinobacteria;c__Coriobacteriia;o__Coriobacteriales;f__Coriobacteriaceae;g__	p__Actinobacteria;c__Actinobacteria;o__Actinomycetales;f__Microbacteriaceae;g__Leucobacter
p__Actinobacteria;c__Coriobacteriia;o__Coriobacteriales;f__Coriobacteriaceae;g__Adlercreutzia	p__Actinobacteria;c__Actinobacteria;o__Actinomycetales;f__Micrococcaceae;g__Rothia
p__Actinobacteria;c__Coriobacteriia;o__Coriobacteriales;f__Coriobacteriaceae;g__Collinsella	p__Actinobacteria;c__Coriobacteriia;o__Coriobacteriales;f__Coriobacteriaceae;g__Olsenella
p__Bacteroidetes;__;__;__;__	p__Actinobacteria;c__Coriobacteriia;o__Coriobacteriales;f__Coriobacteriaceae;g__Slackia
p__Bacteroidetes;c__Bacteroidia;o__Bacteroidales;__;__	p__Bacteroidetes;c__Bacteroidia;o__Bacteroidales;f__[Paraprevotellaceae];g__[Prevotella]
p__Bacteroidetes;c__Bacteroidia;o__Bacteroidales;f__Bacteroidaceae;g__Bacteroides	p__Bacteroidetes;c__Bacteroidia;o__Bacteroidales;f__[Paraprevotellaceae];g__CF231
p__Bacteroidetes;c__Bacteroidia;o__Bacteroidales;f__Prevotellaceae;g__Prevotella	p__Bacteroidetes;c__Bacteroidia;o__Bacteroidales;f__Bacteroidaceae;__
p__Deferribacteres;c__Deferribacteres;o__Deferribacterales;f__Deferribacteraceae;g__Mucispirillum	p__Bacteroidetes;c__Bacteroidia;o__Bacteroidales;f__Porphyromonadaceae;g__Parabacteroides
p__Firmicutes;__;__;__;__	p__Bacteroidetes;c__Bacteroidia;o__Bacteroidales;f__S24-7;g__
p__Firmicutes;c__Bacilli;o__Lactobacillales;f__Enterococcaceae;g__Enterococcus	p__Bacteroidetes;c__Flavobacteriia;o__Flavobacteriales;f__[Weeksellaceae];g__Chryseobacterium
p__Firmicutes;c__Bacilli;o__Lactobacillales;f__Lactobacillaceae;g__Lactobacillus	p__Bacteroidetes;c__Flavobacteriia;o__Flavobacteriales;f__[Weeksellaceae];g__Elizabethkingia
p__Firmicutes;c__Bacilli;o__Lactobacillales;f__Streptococcaceae;g__Streptococcus	p__Bacteroidetes;c__Flavobacteriia;o__Flavobacteriales;f__[Weeksellaceae];g__Wautersiella
p__Firmicutes;c__Clostridia;o__Clostridiales;__;__	p__Bacteroidetes;c__Flavobacteriia;o__Flavobacteriales;f__Flavobacteriaceae;g__
p__Firmicutes;c__Clostridia;o__Clostridiales;f__Eubacteriaceae;g__Pseudoramibacter_Eubacterium	p__Bacteroidetes;c__Sphingobacteriia;o__Sphingobacteriales;f__Sphingobacteriaceae;g__Sphingobacterium
p__Firmicutes;c__Clostridia;o__Clostridiales;f__Lachnospiraceae;__	p__Cyanobacteria;c__4C0d-2;o__YS2;f__;g__
p__Firmicutes;c__Clostridia;o__Clostridiales;f__Ruminococcaceae;__	p__Firmicutes;c__Bacilli;__;__;__
p__Firmicutes;c__Clostridia;o__Clostridiales;f__Ruminococcaceae;g__	p__Firmicutes;c__Bacilli;o__Bacillales;f__Planococcaceae;__
p__Firmicutes;c__Clostridia;o__Clostridiales;f__Ruminococcaceae;g__Anaerotruncus	p__Firmicutes;c__Bacilli;o__Lactobacillales;f__Aerococcaceae;g__Abiotrophia

p__Firmicutes;c__Clostridia;o__Clostridiales;f__Ruminococcaceae;g__Clostridium
p__Firmicutes;c__Clostridia;o__Clostridiales;f__Ruminococcaceae;g__Oscillospira
p__Firmicutes;c__Clostridia;o__Clostridiales;f__Ruminococcaceae;g__Ruminococcus
p__Firmicutes;c__Clostridia;o__Clostridiales;f__Ruminococcaceae;g__Subdoligranulum
p__Firmicutes;c__Clostridia;o__Clostridiales;f__Veillonellaceae;g__Anaerovibrio
p__Firmicutes;c__Clostridia;o__Clostridiales;f__Veillonellaceae;g__Dialister
p__Firmicutes;c__Clostridia;o__Clostridiales;f__Veillonellaceae;g__Megamonas
p__Firmicutes;c__Erysipelotrichi;o__Erysipelotrichales;f__Erysipelotrichaceae;g__[Eubacterium]

p__Proteobacteria;__;__;__;__
p__Proteobacteria;c__Alphaproteobacteria;o__Sphingomonadales;f__Sphingomonadaceae;g__Sphingomonas
p__Proteobacteria;c__Betaproteobacteria;o__Burkholderiales;f__Alcaligenaceae;g__Sutterella
p__Proteobacteria;c__Epsilonproteobacteria;o__Campylobacterales;f__Helicobacteraceae;g__Helicobacter
p__Proteobacteria;c__Gammaproteobacteria;__;__;__
p__Proteobacteria;c__Gammaproteobacteria;o__Enterobacteriales;f__Enterobacteriaceae;__
p__Proteobacteria;c__Gammaproteobacteria;o__Enterobacteriales;f__Enterobacteriaceae;g__Morganella
p__Proteobacteria;c__Gammaproteobacteria;o__Enterobacteriales;f__Enterobacteriaceae;g__Proteus
p__Proteobacteria;c__Gammaproteobacteria;o__Pasteurellales;f__Pasteurellaceae;__
p__Proteobacteria;c__Gammaproteobacteria;o__Xanthomonadales;f__Xanthomonadaceae;g__Stenotrophomonas
p__Spirochaetes;c__[Brachyspirae];o__[Brachyspirales];f__Brachyspiraceae;g__Brachyspira
p__Tenericutes;c__Mollicutes;o__Anaeroplasmatales;f__Anaeroplasmataceae;g__gut

p__Tenericutes;c__Mollicutes;o__RF39;f__;g__

p__Firmicutes;c__Bacilli;o__Lactobacillales;f__Enterococcaceae;g__Vagococcus
p__Firmicutes;c__Bacilli;o__Lactobacillales;f__Leuconostocaceae;__
p__Firmicutes;c__Bacilli;o__Lactobacillales;f__Leuconostocaceae;g__Leuconostoc
p__Firmicutes;c__Bacilli;o__Lactobacillales;f__Leuconostocaceae;g__Weissella
p__Firmicutes;c__Bacilli;o__Lactobacillales;f__Streptococcaceae;g__Lactococcus
p__Firmicutes;c__Clostridia;o__Clostridiales;f__[Mogibacteriaceae];__
p__Firmicutes;c__Clostridia;o__Clostridiales;f__[Mogibacteriaceae];g__
p__Firmicutes;c__Clostridia;o__Clostridiales;f__Clostridiaceae;g__Clostridium
p__Firmicutes;c__Clostridia;o__Clostridiales;f__Clostridiaceae;g__Sarcina

p__Firmicutes;c__Clostridia;o__Clostridiales;f__Lachnospiraceae;g__[Ruminococcus]
p__Firmicutes;c__Clostridia;o__Clostridiales;f__Lachnospiraceae;g__Blautia

p__Firmicutes;c__Clostridia;o__Clostridiales;f__Lachnospiraceae;g__Clostridium
p__Firmicutes;c__Clostridia;o__Clostridiales;f__Lachnospiraceae;g__Coprococcus
p__Firmicutes;c__Clostridia;o__Clostridiales;f__Lachnospiraceae;g__Dorea

p__Firmicutes;c__Clostridia;o__Clostridiales;f__Lachnospiraceae;g__Lachnospira
p__Firmicutes;c__Clostridia;o__Clostridiales;f__Lachnospiraceae;g__Oribacterium
p__Firmicutes;c__Clostridia;o__Clostridiales;f__Lachnospiraceae;g__Roseburia

p__Firmicutes;c__Clostridia;o__Clostridiales;f__Peptococcaceae;g__Peptococcus
p__Firmicutes;c__Clostridia;o__Clostridiales;f__Ruminococcaceae;g__Butyricicoccus
p__Firmicutes;c__Clostridia;o__Clostridiales;f__Ruminococcaceae;g__Faecalibacterium
p__Firmicutes;c__Clostridia;o__Clostridiales;f__Ruminococcaceae;g__Gemmiger
p__Firmicutes;c__Clostridia;o__Clostridiales;f__Veillonellaceae;__
p__Firmicutes;c__Clostridia;o__Clostridiales;f__Veillonellaceae;g__Phascolarctobacterium

p__Firmicutes;c__Clostridia;o__Clostridiales;f__Veillonellaceae;g__Veillonella
p__Firmicutes;c__Erysipelotrichi;o__Erysipelotrichales;f__Erysipelotrichaceae;g__
p__Firmicutes;c__Erysipelotrichi;o__Erysipelotrichales;f__Erysipelotrichaceae;g__Allobaculum
p__Firmicutes;c__Erysipelotrichi;o__Erysipelotrichales;f__Erysipelotrichaceae;g__Bulleidia
p__Firmicutes;c__Erysipelotrichi;o__Erysipelotrichales;f__Erysipelotrichaceae;g__Catenibacterium
p__Firmicutes;c__Erysipelotrichi;o__Erysipelotrichales;f__Erysipelotrichaceae;g__Clostridium
p__Firmicutes;c__Erysipelotrichi;o__Erysipelotrichales;f__Erysipelotrichaceae;g__p-75-a5
p__Firmicutes;c__Erysipelotrichi;o__Erysipelotrichales;f__Erysipelotrichaceae;g__RFN20
p__Fusobacteria;c__Fusobacteriia;o__Fusobacteriales;f__Fusobacteriaceae;__
p__Fusobacteria;c__Fusobacteriia;o__Fusobacteriales;f__Fusobacteriaceae;g__
p__Fusobacteria;c__Fusobacteriia;o__Fusobacteriales;f__Fusobacteriaceae;g__Fusobacterium
p__Fusobacteria;c__Fusobacteriia;o__Fusobacteriales;f__Leptotrichiaceae;g__Leptotrichia
p__Proteobacteria;c__Alphaproteobacteria;o__Caulobacterales;f__Caulobacteraceae;g__Brevundimonas
p__Proteobacteria;c__Alphaproteobacteria;o__Rhizobiales;f__Rhizobiaceae;__
p__Proteobacteria;c__Alphaproteobacteria;o__Rhizobiales;f__Rhizobiaceae;g__Agrobacterium
p__Proteobacteria;c__Alphaproteobacteria;o__Rhizobiales;f__Rhizobiaceae;g__Shinella
p__Proteobacteria;c__Alphaproteobacteria;o__Rhodobacterales;f__Rhodobacteraceae;__
p__Proteobacteria;c__Alphaproteobacteria;o__Rhodospirillales;f__Acetobacteraceae;g__Roseomonas
p__Proteobacteria;c__Alphaproteobacteria;o__Rickettsiales;__;__
p__Proteobacteria;c__Betaproteobacteria;o__Burkholderiales;__;__
p__Proteobacteria;c__Betaproteobacteria;o__Burkholderiales;f__Alcaligenaceae;__
p__Proteobacteria;c__Betaproteobacteria;o__Burkholderiales;f__Burkholderiaceae;g__Lautropia
p__Proteobacteria;c__Betaproteobacteria;o__Burkholderiales;f__Comamonadaceae;__

p__Proteobacteria;c__Betaproteobacteria;o__Bur
kholderiales;f__Comamonadaceae;g__Brachymo
nas
p__Proteobacteria;c__Betaproteobacteria;o__Bur
kholderiales;f__Comamonadaceae;g__Lamproped
ia
p__Proteobacteria;c__Betaproteobacteria;o__Bur
kholderiales;f__Oxalobacteraceae;__
p__Proteobacteria;c__Betaproteobacteria;o__Bur
kholderiales;f__Oxalobacteraceae;g__Massilia
p__Proteobacteria;c__Betaproteobacteria;o__Neis
seriales;f__Neisseriaceae;__
p__Proteobacteria;c__Betaproteobacteria;o__Neis
seriales;f__Neisseriaceae;g__Kingella
p__Proteobacteria;c__Betaproteobacteria;o__Neis
seriales;f__Neisseriaceae;g__Neisseria
p__Proteobacteria;c__Deltaproteobacteria;o__Des
ulfovibrionales;f__Desulfovibrionaceae;__
p__Proteobacteria;c__Deltaproteobacteria;o__Des
ulfovibrionales;f__Desulfovibrionaceae;g__
p__Proteobacteria;c__Deltaproteobacteria;o__Des
ulfovibrionales;f__Desulfovibrionaceae;g__Desul
fovibrio
p__Proteobacteria;c__Epsilonproteobacteria;o__C
ampylobacterales;f__Campylobacteraceae;g__Ca
mpylobacter
p__Proteobacteria;c__Epsilonproteobacteria;o__C
ampylobacterales;f__Helicobacteraceae;g__Flexis
pira
p__Proteobacteria;c__Gammaproteobacteria;o__
Aeromonadales;f__Aeromonadaceae;g__Aeromo
nas
p__Proteobacteria;c__Gammaproteobacteria;o__
Aeromonadales;f__Succinivibrionaceae;g__Anaer
obiospirillum
p__Proteobacteria;c__Gammaproteobacteria;o__
Aeromonadales;f__Succinivibrionaceae;g__Succi
nivibrio
p__Proteobacteria;c__Gammaproteobacteria;o__
Cardiobacteriales;f__Cardiobacteriaceae;__
p__Proteobacteria;c__Gammaproteobacteria;o__
Cardiobacteriales;f__Cardiobacteriaceae;g__
p__Proteobacteria;c__Gammaproteobacteria;o__
Cardiobacteriales;f__Cardiobacteriaceae;g__Card
iobacterium
p__Proteobacteria;c__Gammaproteobacteria;o__
Enterobacteriales;f__Enterobacteriaceae;g__Provi
dencia
p__Proteobacteria;c__Gammaproteobacteria;o__P
asteurellales;f__Pasteurellaceae;g__Aggregatibact
er

	p__Proteobacteria;c__Gammaproteobacteria;o__Pasteurellales;f__Pasteurellaceae;g__Haemophilus
	p__Proteobacteria;c__Gammaproteobacteria;o__Pseudomonadales;f__Moraxellaceae;g__Acinetobacter
	p__Proteobacteria;c__Gammaproteobacteria;o__Pseudomonadales;f__Moraxellaceae;g__Enhydrobacter
	p__Proteobacteria;c__Gammaproteobacteria;o__Pseudomonadales;f__Moraxellaceae;g__Psychrobacter
	p__Proteobacteria;c__Gammaproteobacteria;o__Pseudomonadales;f__Pseudomonadaceae;__
	p__Proteobacteria;c__Gammaproteobacteria;o__Pseudomonadales;f__Pseudomonadaceae;g__Pseudomonas
	p__Proteobacteria;c__Gammaproteobacteria;o__Xanthomonadales;f__Xanthomonadaceae;__
	p__Proteobacteria;c__Gammaproteobacteria;o__Xanthomonadales;f__Xanthomonadaceae;g__Wohlfahrtiimonas
	p__TM7;c__TM7-3;o__;f__;g__
	p__TM7;c__TM7-3;o__I025;f__Rs-045;g__
